# Structural, Physiological and Regulatory Analysis of Maltose Transporter Genes in *Saccharomyces eubayanus* CBS 12357^T^

**DOI:** 10.3389/fmicb.2018.01786

**Published:** 2018-08-10

**Authors:** Anja Brickwedde, Nick Brouwers, Marcel van den Broek, Joan S. Gallego Murillo, Julie L. Fraiture, Jack T. Pronk, Jean-Marc G. Daran

**Affiliations:** Department of Biotechnology, Delft University of Technology, Delft, Netherlands

**Keywords:** yeast, brewing, domestication, gene regulation, wort, transport, *Saccharomyces eubayanus*

## Abstract

*Saccharomyces pastorianus* lager brewing yeasts are domesticated hybrids of *Saccharomyces cerevisiae* and cold-tolerant *Saccharomyces eubayanus*. To understand the contribution of both parental genomes to maltose metabolism in brewing wort, this study focuses on maltose transport in the *S. eubayanus* type strain CBS 12357^T^/FM1318. To obtain complete sequences of the *MAL* loci of this strain, a near-complete genome assembly was generated using the Oxford Nanopore Technology MinION sequencing platform. Except for CHRXII, all sixteen chromosomes were assembled as single contigs. Four loci harboring putative maltose transporter genes (*SeMALT1-4*), located in subtelomeric regions of CHRII, CHRV, CHRXIII, and CHRXVI, were completely resolved. The near-identical loci on CHRV and CHRXVI strongly resembled canonical *S. cerevisiae MAL* loci, while those on CHRII and CHRXIII showed different structures suggestive of gene loss. Overexpression of *SeMALT1-4* in a maltose-transport-deficient *S. cerevisiae* strain restored growth on maltose, but not on maltotriose, indicating maltose-specific transport functionality of all four transporters. Simultaneous CRISPR-Cas9-assisted deletion of only *SeMALT2* and *SeMALT4*, which shared 99.7% sequence identity, eliminated growth of *S. eubayanus* CBS 12357^T^ on maltose. Transcriptome analysis of *S. eubayanus* CBS 12357^T^ established that *SeMALT1* and *SeMALT3*, are poorly expressed in maltose-grown cultures, while *SeMALT2* and *SeMALT4* were expressed at much higher levels than *SeMALT1* and *SeMALT3*, indicating that only *SeMALT2/4* are responsible for maltose consumption in CBS 12357^T^. These results represent a first genomic and physiological characterization of maltose transport in *S. eubayanus* CBS 12357^T^ and provides a valuable resource for further industrial exploitation of this yeast.

## Introduction

*Saccharomyces eubayanus* was first isolated from Nothofagus trees and stromata of *Cyttaria harioti* in North-Western Patagonia (Libkind et al., 2011). Strains of *S. eubayanus* have subsequently been also isolated from locations in North America (Peris et al., 2014), Asia (Bing et al., 2014), and Oceania (Gayevskiy and Goddard, 2016). Initial physiological characterization of the Patagonian *S. eubayanus* strain CBS 12357^T^ revealed that it grows faster than *S. cerevisiae* at temperatures below 10°C (Hebly et al., 2015), shows poor flocculation (Krogerus et al., 2015), and consumes maltose but not maltotriose (Hebly et al., 2015; Gibson et al., 2017).

Isolation and characterization of *S. eubayanus* provided a strong impetus for research on *S. pastorianus* lager brewing yeasts. The hybrid nature of lager yeast genomes was already shown by Southern hybridization (Tamai et al., 1998; Yamagishi et al., 1999); RFLP genotyping, Sanger sequencing (Casaregola et al., 2001; Rainieri et al., 2006), and comparative proteomics (Joubert et al., 2000; Caesar et al., 2007) However, release of the first *S. eubayanus* genome sequence (Libkind et al., 2011) unequivocally established that this cold-tolerant *Saccharomyces* species contributed the non-*cerevisiae* part of *S. pastorianus* genomes (Nakao et al., 2009; Hewitt et al., 2014; Walther et al., 2014; van den Broek et al., 2015) Access to this genome sequence and its updates (Baker et al., 2015; Hebly et al., 2015) proved invaluable for resolving the complex structure of aneuploid *S. pastorianus* genomes. Moreover, access to *S. eubayanus* strains stimulated vigorous research into *de novo* generation of hybrids between *S. cerevisiae* and *S. eubayanus* in the laboratory (Steensels et al., 2014; Hebly et al., 2015; Krogerus et al., 2015, 2017a; Magalhães et al., 2017) This approach has the potential to increase our understanding of the domestication process of lager brewing strains and, moreover, to strongly increase the genetic and phenotypic variety of lager yeast strains available to the brewing industry. *De novo* constructed *S. cerevisiae* × *S. eubayanus* hybrids have been demonstrated to combine advantageous brewing-related properties of both parents (cryo-tolerance, maltotriose utilization, and strong flocculation) and even exhibited best parent heterosis also referred to as hybrid vigor (Steensels et al., 2014; Hebly et al., 2015; Krogerus et al., 2017a,b; Peris et al., 2017). However, generation of new hybrids is, by itself, not sufficient to understand the genetic basis for the exceptional performance of *S. pastorianus* under brewing conditions.

Lager brewing strains of *S. pastorianus* have, over several centuries, been selected for rapid, near-complete fermentation of all-malt brewer's wort fermentable sugars, which typically comprise 60% maltose, 25% maltotriose, and 15% glucose, with trace amounts of fructose (Zastrow et al., 2001). Lager brewing therefore critically depends on the capacity of *S. pastorianus* strains to efficiently take up and ferment the wort α-glucosides maltose and maltotriose. The required maltose fermentation characteristics of *S. pastorianus* strains are conferred by genes originating from each of the parents and from a set that likely arose during its domestication history (e.g., *MTT1*) (Chow et al., 1989; Dietvorst et al., 2005; Salema-Oom et al., 2005; Vidgren et al., 2005; Alves et al., 2008; Vidgren and Londesborough, 2012; Cousseau et al., 2013; Magalhães et al., 2016).

In *S. cerevisiae*, maltose metabolism and the responsible *MAL* genes are well characterized in term of sequence, genetics, regulation and biochemistry. *S. cerevisiae MAL* loci harbor the three key genes essential for maltose utilization, encoding a transcriptional activator (*MALx3*), a maltose permease (*MALx1*) and a maltase (*MAx2*) (Charron et al., 1989). Numbers and identities of *MAL* loci are highly strain dependent, with up to five *MAL* loci (*MAL1, 2, 3, 4*, and *6*) occurring in haploid *S. cerevisiae* genomes. *MAL* loci are typically located in subtelomeric regions, with the structurally identical *MAL1, 2, 3, 4*, and *6* located near telomeres of CHRVII, III, II, XI, and VIII, respectively (Cohen et al., 1985; Dubin et al., 1985; Charron et al., 1989; Chow et al., 1989; Michels et al., 1992; Han et al., 1995; Day et al., 2002b).

Maltose is transported across the *S. cerevisiae* plasma membrane by maltose-proton symport, mediated by Malx1 transporters (Serrano, 1977; van Leeuwen et al., 1992) and, to a lesser extent, by facilitated diffusion (Day et al., 2002a). All *MALx1* genes are highly similar, with the exception of *MAL11* and its allele *AGT1*, whose DNA sequence shows only 57% identity to the other four *MALx1* transporter genes (Han et al., 1995; Vidgren et al., 2009). This sequence difference is accompanied by a difference in substrate range, with Agt1 also being able to transport other α-glucosides, such as trehalose (Plourde-Owobi et al., 1999), sucrose (Basso et al., 2011; Marques et al., 2017) and, importantly for brewing applications, maltotriose (Alves et al., 2008; Vidgren et al., 2009). The *S. cerevisiae* genome harbors two additional maltose permease genes, *MPH2* and *MPH3*, which are located subtelomerically on CHRIV and X, respectively. Although the transport mechanisms of Mph2 and Mph3 have not been experimentally established, both carriers were shown to transport of a range of substrates including glucose, maltose, maltotriose, α-methylglucoside, and turanose (Day et al., 2002a).

In contrast to the wealth of information on *S. cerevisiae*, knowledge on maltose transport in *S. eubayanus* is limited. The type strain *S. eubayanus* CBS 12357^T^ grows on maltose, but not on maltotriose (Hebly et al., 2015). Annotation of its genome sequence revealed four open reading frames sharing similarity with *S. cerevisiae MAL31* (*SeMALT1*; *SeMALT2, SeMALT3*, and *SeMALT4*) (Baker et al., 2015). The hybrid *S. pastorianus* genome harbors two additional maltose transporter gene variants that were not found in either of the reference parental genomes. The first of these, *MTT1*/*MTY1*, shares 90 and 54% DNA sequence identity with *S. cerevisiae MAL31* and *MAL11*, respectively (Dietvorst et al., 2005; Salema-Oom et al., 2005). The second *S. pastorianus*-specific maltose-transporter gene, *SeAGT1*, shared significant identity with *S. cerevisiae AGT1* (85% *ScAGT1*; Vidgren and Londesborough, 2012). *SeAGT1* was unexpectedly found to be located on the *S. eubayanus*-derived CHRVIII-XV, suggesting a *S. eubayanus* origin, despite the absence of similar genes in currently available *S. eubayanus* genome sequences (Nakao et al., 2009; van den Broek et al., 2015) However, genome assembly of an Asian *S. eubayanus* strain (CDFM21L.1; Bing et al., 2014) revealed short (<200 bp) sequences reminiscent of a putative *SeAGT1* gene (Hebly et al., 2015). Both *MTT1/MTY1* and *SeAGT1* were shown to confer low- temperature dependent transport of both maltose and maltotriose (Vidgren et al., 2014). To illustrate the complexity of α-glucoside transport in *S. pastorianus*, the model strain Weihenstephan 34/70 contains all *S. cerevisiae MAL* loci except for *MAL2*, a single *MPH* gene (*MPH2*), as well as all four *S. eubayanus* genes (*MALT1* to *4*) (Nakao et al., 2009; van den Broek et al., 2015) and the two *S. pastorianus*-specific genes *MTT1* and *SeAGT1* (Magalhães et al., 2016). In this *S. pastorianus* background, the *S. cerevisiae* allele of *AGT1* (*MAL11*) carries a nonsense mutation (Vidgren et al., 2009).

Hitherto, no study has systematically investigated the functionality of the individual α-glucoside transporters in *S. pastorianus*. In addition to the complexity of maltose metabolism in *S. pastorianus* strains, genetic analysis is complicated by the limited genetic accessibility of industrial lager brewing yeasts (Bolat et al., 2013). However, availability of the *S. eubayanus* type strain and of its genome sequence offers an alternative approach to fill existing knowledge gaps on transport of wort sugars. The aim of this study was therefore to investigate the contribution of individual putative maltose-transporter (*SeMALT*) genes in *S. eubayanus* CBS 12357^T^. To this end, a new near-complete genome sequence of the strain CBS 12357^T^ was assembled using Oxford Nanopore Technology's MinION long-read sequencing platform. Subsequently, CRISPR-Cas9 gene editing was used to systematically delete the *MALT* genes in *S. eubayanus*. In a complementary approach, all four *S. eubayanus MALT* open reading frames were cloned and constitutively expressed alongside the *S. cerevisiae MAL12* maltase gene in a *S. cerevisiae* strain lacking all maltose utilization genes (*MALx1, MALx2*, and *MALx3*), *MPH1*/*2, SUC2*, and *IMA1*-*5* genes; (Marques et al., 2018). Subsequently, growth of the genetically modified yeast strains was analyzed on different carbon sources. Finally, RNA sequencing was performed on glucose- and maltose-grown cultures to study differential expression of the *S. eubayanus MALT* genes.

## Materials and methods

### Strains and maintenance

*S. eubayanus* strain CBS 12357^T^ (alias FM1318, Libkind et al., 2011) was obtained from the Westerdijk Fungal Biodiversity Institute (Utrecht, the Netherlands, http://www.westerdijkinstitute.nl/). The *S. cerevisiae* strain IMZ616 (Marques et al., 2018) was derived from the CEN.PK lineage (Entian and Kötter, 2007; Salazar et al., 2017). All strains used in this study are listed in Table 1. Stock cultures of *S. eubayanus* and *S. cerevisiae* strains were grown in YPD (10 g L^−1^ yeast extract, 20 g L^−1^ peptone, and 20 g L^−1^ glucose) until late exponential phase, complemented with sterile glycerol to a final concentration of 30% (v/v) and stored at −80°C as 1.5 ml aliquots until further use.

**Table 1:** Strains used in this study.

**Strain**	**Genotype**	**Species**	**References**
CBS 12357^T^/FM1318	*MATa/MATα SeMALT1/SeMALT1 SeMALT2/SeMALT2 SeMALT3/SeMALT3 SeMALT4/SeMALT4*	Se^a^	Libkind et al., 2011
IMK816	*MATa/MATα SemalT1Δ/SemalT1Δ SeMALT2/SeMALT2 SeMALT3/SeMALT3 SeMALT4/SeMALT4*	Se	This study
IMK817	*MATa/MATα SeMALT1/SeMALT1 SemalT2Δ/SemalT2Δ SeMALT3/SeMALT3 SemalT4Δ/SemalT4Δ*	Se	This study
IMK818	*MATa/MAT*α Se*MALT1/SeMALT1 SeMALT2/SeMALT2 SemalT3Δ/SemalT3Δ SeMALT4/SeMALT4*	Se	This study
IMZ616	*MATa ura3-52 HIS3 LEU2 TRP1 mal1Δ::loxP mal2Δ::loxP mal3Δ::loxP mph2Δ::loxP mph3Δ::loxP suc2Δ::loxP-KanMX-loxP ima1Δ ima2Δ ima3Δ ima4Δ ima5Δ* pUDC156 (*Spcas9 URA3 ARS4 CEN6*)	Sc^b^	Marques et al., 2018
IMX1253	*MATa ura3-52 HIS3 LEU2 TRP1 mal1Δ::loxP mal2Δ::loxP mal3Δ::loxP mph2Δ::loxP mph3Δ::loxP suc2Δ::loxP-KanMX-loxP ima1Δ ima2Δ ima3Δ ima4Δ ima5Δ sga1Δ::ScTEF1_*pr*_-SeMALT1-ScCYC1_*ter*_::ScTDH3_*pr*_-ScMAL12- ScADH1_*ter*_* pUDC156 (*Spcas9 URA3 ARS4 CEN6*)	Sc	This study
IMX1254	*MATa ura3-52 HIS3 LEU2 TRP1 mal1Δ::loxP mal2Δ::loxP mal3Δ::loxP mph2Δ::loxP mph3Δ::loxP suc2Δ::loxP-KanMX-loxP ima1Δ ima2Δ ima3Δ ima4Δ ima5Δ sga1Δ:: ScTEF1_*pr*_-SeMALT2-ScCYC1_*ter*::*Sc*_TDH3pr-ScMAL12- ScADH1_*ter*_* pUDC156 (*Spcas9 URA3 ARS4 CEN6*)	Sc	This study
IMX1255	*MATa ura3-52 HIS3 LEU2 TRP1 mal1Δ::loxP mal2Δ::loxP mal3Δ::loxP mph2Δ::loxP mph3Δ::loxP suc2Δ::loxP-KanMX-loxP ima1Δ ima2Δ ima3Δ ima4Δ ima5Δ sga1Δ:: ScTEF1_*pr*_-SeMALT3-ScCYC1_*ter*_::ScTDH3_*pr*_-ScMAL12- ScADH1_*ter*_* pUDC156 (*Spcas9 URA3 ARS4 CEN6*)	Sc	This study
IMX1365	*MATa ura3-52 HIS3 LEU2 TRP1 mal1Δ::loxP mal2Δ::loxP mal3Δ::loxP mph2Δ::loxP mph3Δ::loxP suc2Δ::loxP-KanMX-loxP ima1Δ ima2Δ ima3Δ ima4Δ ima5Δ sga1Δ:: ScTEF1_*pr*_-ScAGT1-ScCYC1_*ter*_::ScTDH3_*pr*_-ScMAL12- ScADH1_*ter*_* pUDC156 (*Spcas9 URA3 ARS4 CEN6*)	Sc	This study

a *S. eubayanus*.

b *S. cerevisiae*.

### Media and cultivation

*S. eubayanus* batch cultures were grown on synthetic medium (SM) containing 3.0 g L^−1^ KH_2_PO_4_, 5.0 g L^−1^ (NH_4_)_2_SO_4_, 0.5 g L^−1^ MgSO_4_, 7 H_2_O, 1 mL L^−1^ trace element solution, and 1 mL L^−1^ vitamin solution (Verduyn et al., 1992). The pH was set to 6 with 2 M KOH prior to autoclaving at 120°C for 20 min. Vitamin solutions (Verduyn et al., 1992) were sterilized by filtration and added to the sterile medium. Concentrated sugar solutions were autoclaved at 110°C for 20 min and added to the sterile flasks to give a final concentration of 20 g L^−1^ carbon source [glucose (SMG), maltose (SMM) or maltotriose (SMMt)]. *S. cerevisiae* batch cultures were grown on SM supplemented with 150 mg L^−1^ uracil to compensate for loss of plasmid pUDC156 that carried the *cas9* endonuclease gene, and supplemented with 20 g L^−1^ carbon source [glucose (SM_u_G), maltose (SM_u_M) or maltotriose (SM_u_Mt)]. All batch cultures were grown in 500 mL shake flasks with a working volume of 100 mL. The cultures were inoculated at an initial OD_660nm_ of 0.1 and incubated under an air atmosphere and shaken at 200 rpm and at 20°C in a New Brunswick™ Innova44 incubator (Eppendorf Nederland B.V, Nijmegen, The Netherlands).

Selection of the *S. eubayanus* strains transformed with plasmids pUDP062 (gRNA_*SeMALT*1_), pUDP063 (gRNA_*SeMALT*2_), and pUDP064 (gRNA_*SeMALT*3_) was carried out on a modified SMG medium, in which (NH_4_)_2_SO_4_ was replaced by 5 g.L^−1^ K_2_SO_4_ and 10 mM acetamide (SM_Ace_G) (Solis-Escalante et al., 2013). SM- based solid medium contained 2% Bacto Agar (BD, Franklin Lakes, NJ). Selection of *S. cerevisiae* integration strains was carried out on SM_Ace_G. For plasmid propagation, *E. coli* XL1-Blue-derived strains (Agilent Technologies, Santa Clara, CA) were grown in Lysogeny Broth medium (LB, 10 g L^−1^ tryptone, 5 g L^−1^ yeast extract, 5 g L^−1^ NaCl) supplied with 100 mg L^−1^ ampicillin.

### Plasmid and strain construction

#### Plasmid construction

Guide-RNA (gRNA) sequences for deletion of *SeMALT1, SeMALT2/T4* and *SeMALT3* were designed following the guiding principles recommended in Gorter de Vries et al. (2017). The DNA sequences encoding these gRNAs were synthesized at GeneArt (Thermo Fisher Scientific, Waltham, MA) and were delivered in pUD631, pUD632, and pUD633 respectively (Table 2). The gRNA spacer sequences (Se*MALT1* 5′ATTCCAAACGACAATAAAGA3′, Se*MALT2/T4* 5′-TACAGGAGAATGGGAGATTT-3′ and Se*MALT3* 5′- GTTTTCAAAGCTTGCAGAAG-3′) and the structural gRNA sequence were flanked at their 5′ ends by the Hammerhead ribozyme (HH) and at their 3′ ends by the Hepatitis Delta Virus ribozyme (HDV) (Gao and Zhao, 2014). The HH-gRNA-HDV fragment was flanked on both ends with a BsaI site for further cloning (Gorter de Vries et al., 2017; Juergens et al., 2018). In the next step, the gRNAs were transferred into the pUDP004 plasmid (Gorter de Vries et al., 2017), which enables combined expression of the gRNA cassette and of *Spcas9*^D147YP411T^ (Bao et al., 2015). The plasmid pUDP062, expressing gRNA_*SeMALT*1_ was constructed in a one-pot reaction by digesting pUDP004 and pUD631 using BsaI and ligating with T4 ligase. Similarly, pUDP063, expressing gRNA_*SeMAT*2/*T*4_ and *Spcas9*^*D*147*YP*411*T*^ was assembled from pUDP004 and pUD632. The plasmid pUDP064 expressing gRNA_*SeMALT*3_ and *Spcas9*^*D*147*YP*411*T*^ was assembled from pUDP004 and pUD633. Correct assembly of pUDP062-064 was verified by restriction analysis with SspI and PdmI.

**Table 2:** Plasmids used in this study.

**Name**	**Relevant characteristics**	**Origin**
p426-TEF-amdS	ori (ColE1) *bla* 2μ amdSYM *TEF1_*pr*_*-*CYC1_*ter*_*	Solis-Escalante et al., 2013
pUDP004	ori (ColE1) *bla* panARSopt amdSYM Sc*TDH3_*pr*_*-BsaI-BsaI-Sc*CYC1_*ter*_ AaTEF1_*pr*_*-*Spcas9*^D147YP411T^-*ScPHO5_*ter*_*	Gorter de Vries et al., 2017
pUDP052	ori (ColE1) *bla* panARSopt amdSYM Sc*TDH3_*pr*_*- *gRNA_*SGA*1_*-Sc*CYC1_*ter*_ AaTEF1_*pr*_*-*Spcas9*^D147YP411T^-*ScPHO5_*ter*_*	This study
pUDP062	ori (ColE1) *bla* panARSopt amdSYM Sc*TDH3_*pr*_*- *gRNA_*SeMALT*1_*-Sc*CYC1_*ter*_ AaTEF1_*pr*_*-*Spcas9*^D147YP411T^-*ScPHO5_*ter*_*	This study
pUDP063	ori (ColE1) *bla* panARSopt amdSYM Sc*TDH3_*pr*_*- *gRNA_*SeMALT*2/*T*4_*-Sc*CYC1_*ter*_ AaTEF1_*pr*_*-*Spcas9*^D147YP411T^-*ScPHO5_*ter*_*	This study
pUDP064	ori (ColE1) *bla* panARSopt amdSYM Sc*TDH3_*pr*_*- *gRNA_*SeMALT*3_*-Sc*CYC1_*ter*_ AaTEF1_*pr*_*-*Spcas9*^D147YP411T^-*ScPHO5_*ter*_*	This study
pUDE044	ori (ColE1) *bla* 2μ *ScTDH3_*pr*_*-*ScMAL12*-*ScADH1_*ter*_ URA3*	Basso et al., 2011
pUD479	ori (ColE1) *bla* 2μ amdSYM *ScTEF1_*pr*_*-*SeMALT1*-*ScCYC1_*ter*_*	This study
pUD480	ori (ColE1) *bla* 2μ amdSYM *ScTEF1_*pr*_*-*SeMALT2/4*-*ScCYC1_*ter*_*	This study
pUD481	ori (ColE1) *bla* 2μ amdSYM *ScTEF1pr*-*SeMALT3*-*ScCYC1ter*	This study
pUD445	ori (ColE1) *bla* 2μ amdSYM *ScTEF1pr*-*ScAGT1*-*ScCYC1ter*	This study
pUDR119	ori (ColE1) *bla* 2μ AmdSYM *SNR5*2_pr−_gRNA*_*SGA*1_*-*SUP4_*ter*_*	van Rossum et al., 2016

The coding regions of *SeMALT1, SeMALT2* and *SeMALT3* were amplified from CBS 12357^T^ genomic DNA with Phusion High-Fidelity DNA polymerase (ThermoFisher Scientific), according to the supplier's instructions with primers pairs 10491/10492, 10632/10633, and 10671/10672 (Table S5), respectively. The coding sequence of *ScAGT1* was amplified from CEN.PK113-7D genomic DNA with Phusion High-Fidelity DNA polymerase (ThermoFisher Scientific), according to the supplier's instructions with primers pairs 9940/9941. Each primer carried a 40 bp extension complementary to the plasmid backbone of p426-TEF-amdS (Solis-Escalante et al., 2013; Marques et al., 2018), which was PCR amplified using Phusion High-Fidelity DNA polymerase (ThermoFisher Scientific) and primers 7812 and 5921 (Table S5). p426-TEF-amdS is an expression plasmid that harbors the promoter of the translational elongation factor EF-1 alpha (*TEF1*) of *S. cerevisiae*. Each *SeMALT* fragment was assembled with the p426-TEF-amdS backbone fragment using NEBuilder HiFi DNA Assembly (New England Biolabs, Ipswich, MA), resulting in plasmids pUD479 (*SeMALT1*), pUD480 (*SeMALT2/T4*), pUD481 (*SeMALT3*), and pUD445 (*ScAGT1*) (Table 2).

#### Strains construction

**Figure 1:**
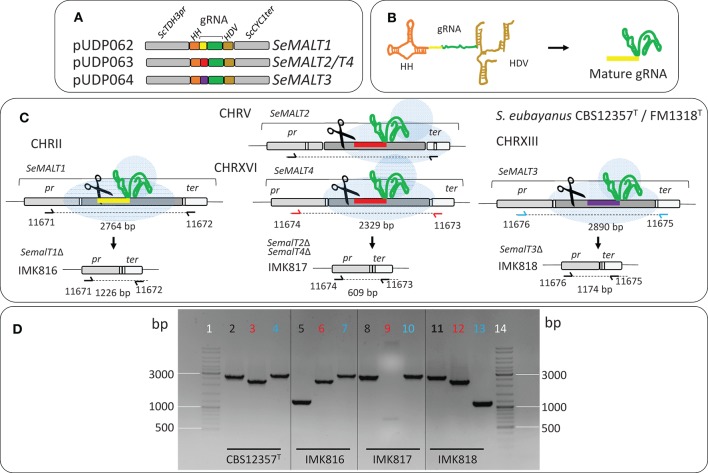
Deletion of *SeMALT* genes using CRISPR-Cas9-assisted genome editing in *S. eubayanus* CBS 12357^T^. **(A)** Representation of the gRNA expression cassette in pUDP062, pUDP063, and pUDP064. gRNAs targeting either *SeMALT1, SeMALT2/T4* or *SeMALT3* were flanked by a 5′ hammerhead ribozyme (HH, orange) and a 3′ hepatitis-δ virus ribozyme (HD, bronze). These constructs were expressed from the RNA polymerase II *ScTDH3* promoter and the *ScCYC1* terminator. **(B)** Upon ribozyme self-cleavage, a mature gRNA comprising the Se*MALT* guiding spacer (yellow) and the constant structural gRNA fragment (green) is released. **(C)** Schematic representation of Se*MALT* gene editing upon transformation of pUDP062 or pUDP063 or pUDP064 into *S. eubayanus* CBS 12357^T^. Primers used for verification of transformants from transformation are indicated together with the size of the expected PCR products. **(D)** Validation of transformants derived from transformations of *S. eubayanus* CBS 12357^T^ with either pUDP062, pUDP063 or pUDP064 in presence of the corresponding 120 bp repair DNA fragments. Lanes 1 and 14 GeneRuler DNA Ladder Mix (Thermo Fischer Scientific). Lanes 2, 5, 8, and 11 fragments amplified with primers 11671 and 11672 black label). Lanes 3, 6, 9, 12 fragments amplified with primers 11674 and 11673 (red label). Lanes 4, 7, 10, and 13 fragments amplified with primers 11676 and 11675 (blue label) from genomic DNA from CBS 12357^T^ (Lanes 2, 3, and 4), from IMK816 (*SemalT1*Δ) (Lanes 5, 6, and 7), from IMK817 (*SemalT2*Δ*/ SemalT4*Δ) (Lanes 8, 9, and 10), and from IMK818 (*SemalT3*Δ) (Lanes 11, 12, and 13).

*S. eubayanus* IMK816 (Se*malT1*Δ) was constructed by transforming CBS 12357^T^ by electroporation (Gorter de Vries et al., 2017) with 200 ng of pUDP062 and 1 μg of 120 bp repair fragment obtained by mixing an equimolar amount of primers 11850 and 11851 (Table S5) (Mans et al., 2015) (Figure 1). As control, the same transformation was performed without including the repair DNA fragment. Transformants were selected on SM_Ace_G plates. Strain IMK817 (*SemalT2*Δ *SemalT4*Δ) and IMK818 (*SemalT3*Δ) were constructed in the same way. The *SeMALT2/T4* deletion was constructed by co-transforming pUDP063 and a repair DNA fragment formed by primers 11328 and 11329, while the *SeMALT3* deletion involved pUDP064 and a repair DNA formed by primers 11330 and 11331 (Table S5). Deletion of *SeMALT1, SeMALT2/T4*, and *SeMALT3* was verified by diagnostic PCR, using primers pairs 11671/11672, 11673/11674, and 11675/11676 (Table S5), respectively (Figure 1C). Prior to storing at −80°C, transformants were successively streaked on SM_Ace_G and YPD plates. The genotype was verified after each plating round with the primers pairs mentioned above.

**Figure 2:**
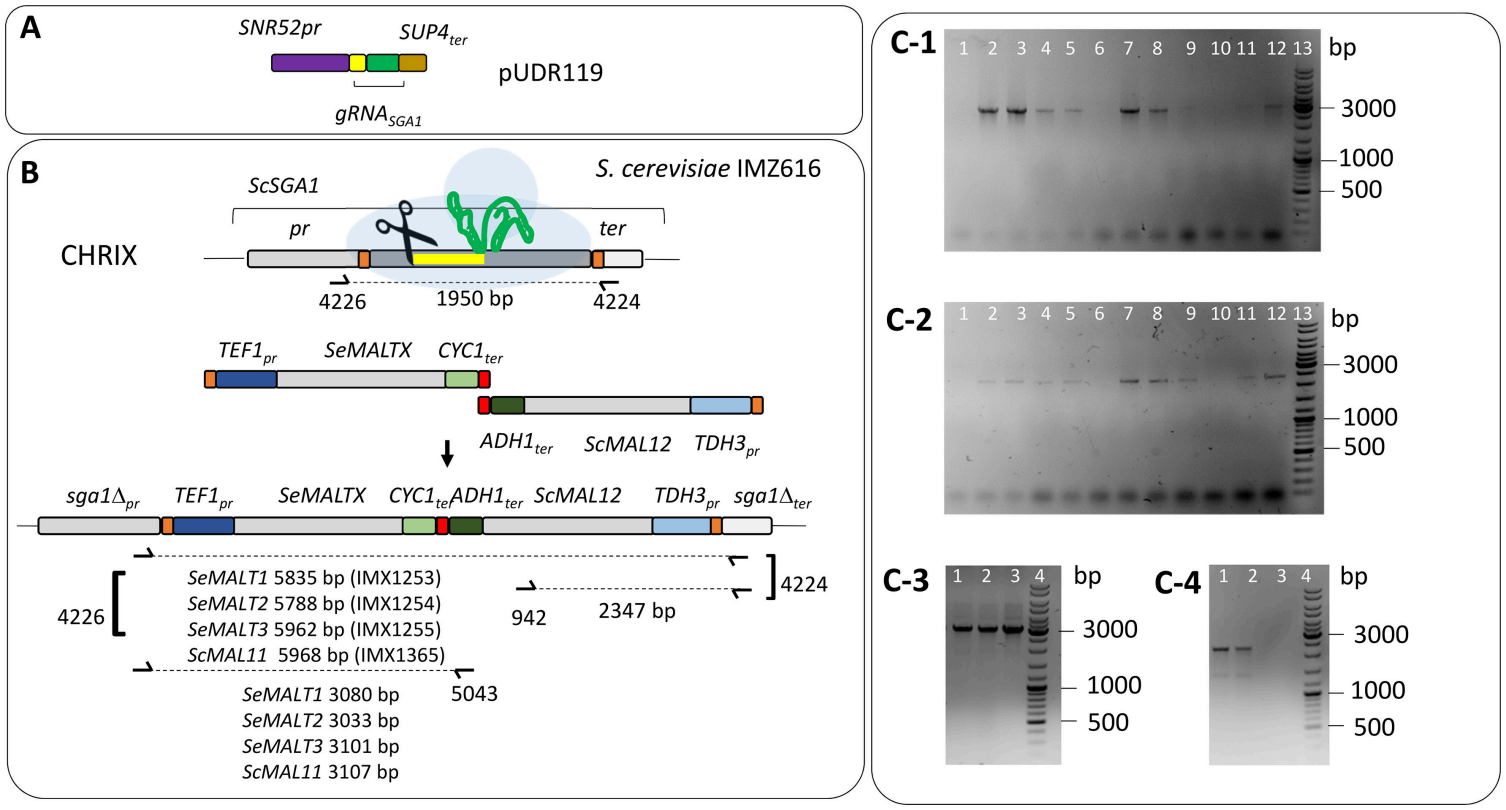
Integration of *S. eubayanus* CBS 12357^T^ maltose transporter genes at the *ScSGA1* locus of *S. cerevisiae* IMZ616 (*mal1*Δ *mal2*Δ *mal3*Δ *mph2*Δ *mph3*Δ*::suc2*Δ *ima1*Δ *ima2*Δ *ima3*Δ *ima4*Δ *ima5*Δ *Spcas9*) (Marques et al., 2018). **(A)** Integration at the Sc*SGA1* locus by Cas9-assisted genome editing. The Cas9-targeting gRNA was expressed from pUDR119 (van Rossum et al., 2016). **(B)** Schematic representation of the integration of *SeMALT* expression cassettes at the *ScSGA1* locus. Upon cleavage, the Cas9-induced double strand break was repaired by the two co-transformed fragments harboring a transporter gene expression cassette and the *S. cerevisiae* maltase gene *MAL12*, respectively. Primers used for verification of transformants from transformation are indicated together with the size of the expected PCR products. Integration of *SeMALT1, SeMALT2, SeMALT3* or *ScMAL11* resulted in *S. cerevisiae* strains IMX1253, IMX1254, IMX1255, and IMX1365 respectively. **(C)** Validation of the *S. cerevisiae* IMX1253, IMX1254, IMX1255, and IMX1365. **(C-1)** Lane 13 GeneRuler DNA Ladder Mix (Thermo Fischer Scientific). Lanes 1 to 12 fragments amplified with primers 4226 and 5043. Lanes 1, 2, 3, and 4 fragments amplified from clones transformed with *SeMALT1*. The strain corresponding to lane 3 was renamed IMX1253. Lanes 5, 6, 7, and 8 fragments amplified from clones transformed with *SeMALT2*. The strain corresponding to lane 7 was renamed IMX1254. Lanes 9, 10, 11, and 12 fragments amplified from clones transformed with *SeMALT3*. The strain corresponding to lane 12 was renamed IMX1255. **(C-2)** Lane 13 GeneRuler DNA Ladder Mix (Thermo Fischer Scientific). Lanes 1 to 12 fragments amplified with primers 942 and 4224. Lanes 1, 2, 3, and 4 fragments amplified from clones transformed with *SeMALT1*. Lane 3 corresponds to IMX1253. Lanes 5, 6, 7, and 8 fragments amplified from clones transformed with *SeMALT2*. Lane 7 corresponds to IMX1254. Lanes 9, 10, 11, and 12 fragments amplified from clones transformed with *SeMALT3*. Lane 12 corresponds to IMX1255. **(C-3)** Lanes 1 to 3 fragments amplified with primers 4226 and 5043. Lanes 1, 2, 3, and 4 fragments amplified from clones transformed with *ScMAL11*. The strain corresponding to lane 3 was renamed IMX1365. Lane 4 GeneRuler DNA Ladder Mix (Thermo Fischer Scientific). **(C-4)** Lanes 1 to 4 fragments amplified with primers 942 and 4224. Lanes 1, 2, and 3 fragments amplified from clones transformed with *ScMAL11*. Lane 3 corresponds to IMX1365. Lane 4 GeneRuler DNA Ladder Mix (Thermo Fischer Scientific).

*S. cerevisiae* IMZ616 [*mal1*Δ *mal2*Δ *mal3*Δ *mph2*Δ *mph3*Δ *suc2*Δ *ima1*Δ *ima2*Δ *ima3*Δ *ima4*Δ *ima5*Δ pUDC156 (*Spcas9 URA3 ARS4 CEN6*)], which cannot grow on α-glucosides (Marques et al., 2018) was used as a host to test the functionality of individual *S. eubayanus* (putative) maltose transporter genes. *S. cerevisiae* IMX1253 was constructed by integrating the *S. cerevisiae* maltase gene *ScMAL12* and the *SeMALT1* transporter gene at the *ScSGA1* locus of strain IMZ616 (Figure 2). The *ScSGA1* gene encodes an intracellular sporulation-specific glucoamylase (Yamashita and Fukui, 1985) that is not expressed during vegetative growth (Knijnenburg et al., 2009). This integration site was shown suitable for expression of single or multiple genes as previously demonstrated in Mans et al. (2015), Kuijpers et al. (2016), Verhoeven et al. (2017), and Bracher et al. (2018) The fragment containing *ScMAL12* was PCR amplified using Phusion High-Fidelity DNA polymerase (Thermo FisherScientific) from pUDE044 (Basso et al., 2011) with primers 9596 and 9355, which included a 5′ extension homologous to the upstream region of the *S. cerevisiae SGA1* locus and an extension homologous to the co-transformed transporter fragment, respectively. The DNA fragment carrying the *S. eubayanus SeMALT1* maltose symporter was PCR amplified from pUD479 using primers 9036 and 9039, which included a 5′ extension homologous to the co-transformed transporter fragment and an extension homologous to the downstream region of the *S. cerevisiae SGA1* locus, respectively. To facilitate integration in strain IMZ616, the two PCR fragments were co-transformed with plasmid pUDR119, which expressed a gRNA targeting Sc*SGA1* (spacer sequence: 5′-ATTGACCACTGGAATTCTTC-3′) (van Rossum et al., 2016) (Figure 2A). The plasmid and repair fragments were transformed using the LiAc protocol (Gietz and Schiestl, 2007) and transformed cells were plated on SM_Ace_G. Correct integration was verified by diagnostic PCR with primers pairs 4226/5043 and 942/4224 (Figure 2, Table S5). Strains *S. cerevisiae* IMX1254, IMX1255, and IMX1365 were constructed following the same principle, but instead of using pUD479 to generate the transporter fragment, pUD480 pUD481 and pUD445 were used to PCR amplify Se*MALT2/T4*, Se*MALT3*, and *ScAGT1* respectively. Correct integration was verified by diagnostic PCR with primers pairs 4226/5043 and 942/4224 (Figure 2, Table S5). All PCR-amplified gene sequences were Sanger sequenced (Baseclear, Leiden, The Netherlands).

### Genome sequencing

#### Illumina sequencing

Genomic DNA from *S. eubayanus* CBS 12357^T^ was isolated as previously described in van den Broek et al. (2015). Paired-end sequencing (2-fold 150 bp) was performed on a 350 bp PCR-free insert library using Illumina HiSeq2500 (San Diego, CA) by Novogene (HK) Company Ltd (Hong Kong, China) with a sample size of 3.2 Gbase. Sequence data are available at NCBI under Bioproject accession number PRJNA450912.

#### MinION sequencing

For Nanopore sequencing, a 1D sequencing library (SQK-LSK108) was prepared according to the manufacturer's recommendation and loaded onto an FLO-MIN106 (R9.4) flow cell, connected to a MinION Mk1B unit (Oxford Nanopore Technology, Oxford, United Kingdom). MinKNOW software (version 1.5.12; Oxford Nanopore Technology) was used for quality control of active pores and for sequencing. Raw files generated by MinKNOW were base called using Albacore (version 1.1.0; Oxford Nanopore Technology). Reads, in fastq format, with a minimum length of 1,000 bp were extracted, yielding 3.26 Gb of sequence with an average read length of 8.07 kb. Sequencing data are available at NCBI under Bioproject accession number PRJNA450912.

#### *De novo* assembly

*De novo* assembly of the Oxford Nanopore MinION dataset was performed using Canu (v1.4, setting: genomesize = 12 m; Koren et al., 2017). Assembly correctness was assessed using Pilon (Walker et al., 2014) and further correction “polishing” of sequencing/assembly errors was performed by aligning Illumina reads with BWA (Li and Durbin, 2010) using correction of only SNPs and short indels (–fix bases parameter). Genome assembly gene annotation was performed with the MAKER2 annotation pipeline (version 2.31.9) (Holt and Yandell, 2011) using SNAP (version 2013–11-29) (Korf, 2004) and Augustus (version 3.2.3) (Stanke et al., 2006) as *ab initio* gene predictors. *S. cerevisiae* S288C EST and protein sequences were obtained from SGD (Saccharomyces Genome Database, http://www.yeastgenome.org/) and were aligned using BLASTX on the obtained polished sequence assembly (BLAST version 2.2.28+) (Camacho et al., 2009). Predicted translated protein sequences of the final gene model were aligned to the *S. cerevisiae* S288C protein Swiss-Prot database using BLASTP (http://www.uniprot.org/). Custom-made Perl scripts were used to map systematic names to the annotated gene names. Error rates in the nanopore-sequencing data were estimated from the q score (Phred scaled) per read, as calculated by the base caller Albacore (version 1.1.0) (Oxford Nanopore Technology). Average q score was used to calculate the error *P* = 10^q/10^.

### Transcriptome analysis

#### RNA isolation

*S. eubayanus* CBS 12357^T^ was grown in either SMG or SMM until mid-exponential phase (OD_660nm_ of 12.5). Culture samples corresponding to ca. Two Hundred and Forty Milligram of biomass wet weight were directly quenched in liquid nitrogen. The resulting frozen pellet was gently thawed on ice and spun down at 4700 × g for 5 min at 0°C. Pellets were then resuspended in 1.2 mL of ice-cold AE buffer (50 mM sodium acetate and 10 mM EDTA, pH 5.0), followed by addition of 1.2 mL of acid phenol/chloroform/isoamyl alcohol mix and 0.12 mL 10% sodium dodecyl sulfate. The resulting mix was vortexed for 30 s and incubated for 5 min at 65°C. After homogenizing for 30 s by vortexing, 800 μL aliquots were distributed in RNase-free screw-cap tubes (Tai et al., 2005). After centrifugation (15 min at 10,000 × g), the aqueous phase was transferred to a new tube containing 0.4 mL of acid phenol/chloroform. The mix was vortexed for 30 s, centrifuged (15 min at 10,000 × g) and the aqueous phase was again transferred to a new tube. RNA was then ethanol precipitated and re-suspended in RNAse-free water. Prior to cDNA synthesis, purity, concentration, and integrity of the RNA in the samples was assessed with the Nanodrop (Thermo Fisher Scientific), Qubit (Thermo Fisher Scientific), and Tapestation 220 with RNA Screen Tape (Agilent Technologies), respectively, according the manufacturers' recommendations. cDNA libraries were prepared using the TruSeq RNA V2 kit (Illumina) and sequenced on HISeq 2500 (Illumina) at Novogene (HK) Company Ltd (Hong Kong, China).

### Transcriptome analysis

Libraries with 300 bp insert size were paired end sequenced (150 bp). Duplicate biological samples were processed, generating an average sequence quantity of 23.7 M reads per sample. Reads were aligned to the Oxford Nanopore CBS 12357^T^ reference assembly using a two-pass STAR (Dobin and Gingeras, 2016) procedure. In the first pass, splice junctions were assembled and used to inform the second round of alignments. Introns between 15 and 4,000 bp were allowed, and soft clipping was disabled to prevent low-quality reads from being spuriously aligned. Ambiguously mapped reads were removed from the dataset. Expression level for each transcript were quantified using htseq-count (Anders et al., 2015) in union mode. Fragments per kilo-base of feature (gene) per million reads mapped (FPKM) values were calculated by “Applying the rpkm method” from the edgeR package (Robinson et al., 2010; McCarthy et al., 2012) Differential expression analysis was performed using DESeq (Anders et al., 2013). Transcript data can be retrieve at the Genome Omnibus Database (GEO: https://www.ncbi.nlm.nih.gov/geo/) under accession number: GSE117246.

### Analytical methods

Optical densities of yeast cultures were measured with a Libra S11 spectrophotometer (Biochrom, Cambridge, UK) at a wavelength of 660 nm. Biomass dry weight was measured by filtering 10-mL culture samples over pre-weighed nitrocellulose filters with a pore size of 0.45 μm. Filters were washed with 10 mL water, dried in a microwave oven (20 min at 350 W) and reweighed. Each measurement was performed in duplicate. For glucose, maltose, maltotriose, and ethanol analysis, culture samples were centrifuged 5 min at 10,000 g and supernatants were analyzed by high-performance liquid chromatography (HPLC) analysis on an Agilent 1260 HPLC equipped with a Bio-Rad HPX 87 H column (Bio-Rad, Hercules, CA). Elution was performed at 65°C with 5 mM H_2_SO_4_ at a flow rate of 0.8 mL min^−1^. Detection was by means of an Agilent refractive-index detector and an Agilent 1260 VWD detector.

#### Viability measurements using fluorescence-assisted cell sorting

Cultures were analyzed on a BD FACSAria™ II SORP Cell Sorter (BD Biosciences, Franklin Lakes, NJ) equipped with 355, 445, 488, 561, and 640 nm lasers and a 70 μm nozzle, and operated with filtered FACSFlow™ (BD Biosciences). Correct cytometer performance was evaluated prior to each experiment by running a CST cycle with corresponding CS&T Beads (BD Biosciences). Drop delay for sorting was determined by running an Auto Drop Delay cycle with Accudrop Beads (BD Biosciences). Morphology of the cells was analyzed by plotting forward scatter (FSC) against side scatter (SSC). Ninety-Six single cells were sorted onto 96-well format Nunc omnitray (Thermo Scientific) plates containing YPD agar using a “single cell” sorting mask, corresponding to a yield mask of 0, a purity mask of 32 and a phase mask of 16. Viability was measured as the average percentage of sorted cells able to form a colony after 48 h incubation at 30°C on three triplicate plates.

## Results

### A high-quality *S. eubayanus* genome assembly with 330 kb of previously unexplored sequence including four *MAL* loci

Owing to advances in genome sequencing technology, the quality of genome sequence data of *S. eubayanus* CBS 12357^T^/FM1318 has gradually improved (Libkind et al., 2011; Baker et al., 2015; Hebly et al., 2015; Okuno et al., 2016) The currently available reference sequence is based on second generation sequencing technology (Illumina generated data), obtained from libraries with different insert sizes that were co-assembled into a 11.66 Mb genome, comprising 144 contigs forming 22 scaffolds. While representing an important resource for research on *S. eubayanus* and *S. pastorianus*, this most advanced draft genome sequence is incomplete (Baker et al., 2015). In particular multiple repeated regions, such as subtelomeric regions, are not yet fully resolved due to limitations of short-read sequencing technology. In total, approximately 122 kb of the scaffolded genome remain undefined.

**Figure 3:**
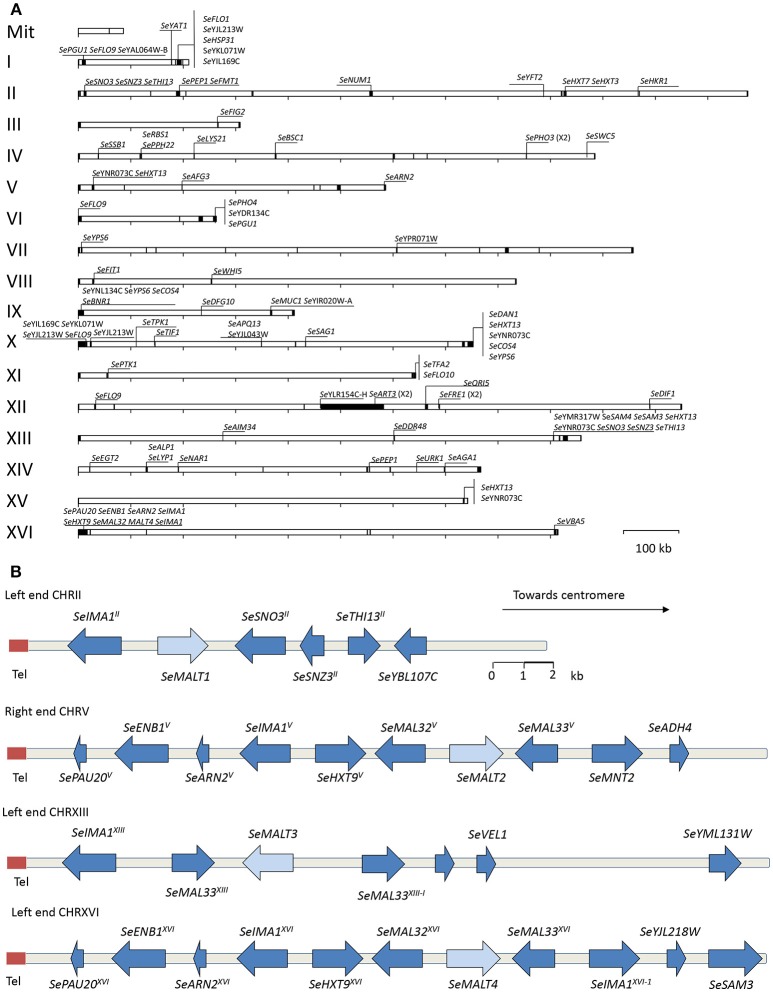
High-quality *S. eubayanus* CBS12357^T^ genome assembly with 330 kb of unexplored sequence including four MAL loci. **(A)** Representation of the assembled *S. eubayanus* chromosomes, the black boxes denote newly added sequences. New annotated open reading frames and gene entries modified relative to the earlier draft genome (Baker et al., 2015) often leading to a redefinition of start and stop codons. **(B)** Organization of subtelomeric regions including maltose metabolism genes. Arrows denote the direction of transcription. The label “Tel” indicates the position of the telomere. The *SeMALT* genes are indicated in light blue. The gene and interval sizes are approximately to scale.

To generate a near-complete, chromosome-level *de novo* assembly of *S. eubayanus* CBS 12357^T^, we used long-read sequencing with third-generation single-molecule technology (Oxford Nanopore Technology MinION platform). A single flow cell was used to generate 3.3 Gb of sequence reads, with an average read length of 8 kb and an estimated average error rate of 9.6%. These data represented a genome coverage of 135 fold of the estimated diploid genome size (24 Mb). An assembly exclusively based on the MinION reads was generated with the Canu program (Koren et al., 2017). This assembly yielded 19 contigs, which is 8-fold fewer than obtained in the short-read-only assembly of the latest CBS 12357^T^ draft genome (Baker et al., 2015). In the MinION-based assembly, the mitochondrial genome and all chromosomes except for CHRXII were assembled as single contigs. CHRXII was manually reconstructed by joining three contigs, with a 1,000 N residues gaps introduced between the contigs. The sequence discontinuity was caused by the inability of the assembly software to handle the highly repetitive DNA organization of the rDNA locus (Figure 3). This approach yielded a nearly complete 11.9 Mb genome assembly.

Prior to annotation, the assembly based on MinION sequencing data was “polished” with additional Illumina sequencing data using Pilon (Walker et al., 2014). *In fine* this polished genome assembly included 330 kb of sequence which were not assembled in the previous genome assembly. With the exception of a region on chromosome XII that corresponded to a partially reconstructed rDNA locus, additional sequences were mainly located in the subtelomeric regions (Figure 3A). A total of 5,444 ORFs were annotated, including 41 previously unassembled ORFs (Baker et al., 2015). Additionally, 60 ORFs were modified relative to the earlier draft genome, often leading to a redefinition of start and stop codons (Figure 3A, Table S6). For 65 of the 101 new genes, a paralog or an identical copy of the gene had already been assembled at a different location in the previous assembly. Gene ontology analysis using Fischer's Exact Test revealed an overrepresentation among the new genes of three GO categories related to sugar transport and cell wall (Table 3). This is not that surprising since, subtelomeres are acknowledged to be the major chromosomal regions involved functional evolution as they are sites for large rearrangement in term of structure, gene content and copy number (not only gain but also loss-of-function variants; Brown et al., 2010; Bergström et al., 2014).

**Table 3:** Overrepresented GO functional categories among 101 newly identified genes in a MinION-sequencing based *S. eubayanus* CBS 12357 genome assembly annotation.

**GO**	**Function**	**Genes**	*P*-value^*^
GO:0031225	Anchored component of membrane (15)	*SeFLO1, SeFLO9* (X4), Se*FLO10, SeFIG2, SeYPS6* (X3), Se*SAG1, SeDAN1, SeEGT2, SeAGA1*,	1.78E−10
GO:0005618	Cell wall (15)	*Se*YIL169C	7.04E−09
GO:0008645	Hexose transport (8)	*SeHXT7, SeHXT3, Se HXT13* (X4), Se*MAL31/SeMALT4, SeHXT9*	4.77E−4

* *Enrichment of functional categories was assessed by Fisher's exact test using Bonferroni correction*.

Four subtelomeric regions harbored complete sequences of putative maltose transporters. Two of these, which contained *SeMALT1* and *SeMALT3*, showed structural features that differed from those of canonical *S. cerevisiae MAL* loci. The CHRII locus only contained a transporter gene (Se*MALT1*) while the CHRXIII “*SeMAL* locus” consisted of a transporter gene (Se*MALT3*) flanked by two non-identical genes that strongly resembled the *S. cerevisiae* regulator genes *MAL33* and *MAL63* (Figure 3B). In contrast, the *S. eubayanus MAL* loci on CHRV and CHRXVI showed the same organization as the well described *S. cerevisiae MAL* loci. Starting from their telomeric ends, they contained a maltase gene (*SeMAL32*), followed by the transporter gene (*SeMALT2* on CHRV and *SeMALT4* on CHRXVI), which shared a bi-directional promoter with the maltase gene, and a *MAL* regulator gene (Figure 3B, Figure S8). Similarity between the right-arm CHRV and left-arm CHRXVI subtelomeric regions extended beyond the *SeMAL* genes, with a sequence identity of 94% and shared gene synteny over a 20 kb region (Figure 3B). The fully assembled *SeMALT4* gene shared 99.7% identity with *SeMALT2*, from which it differed by only five nucleotides. None of these five nucleotide variations affected the predicted amino acid sequence of the encoded transporters.

### Systematic deletion of *MALT* genes revealed that *SeMALT2*/*SeMALT4* are essential for growth maltose

To explore the contribution of the four *S. eubayanus MALT* genes to maltose consumption, deletion strains were constructed. Because the high sequence similarity of *SeMALT2* and *SeMALT4* complicated individual deletion of these genes, three strains were constructed with either a single deletion of *SeMALT1* or *SeMALT3* or a double deletion of *SeMALT2* and *SeMALT4* (Figure 1). The option offered by CRISPR-Cas9 to simultaneously delete of multiple gene copies in a single transformation step (Mans et al., 2015) is especially helpful in diploid strains such as *S. eubayanus* CBS 12357^T^. To explore the use of this methodology in *S. eubayanus*, we used a broad-host-range yeast plasmid for co-expression of *Spcas9* and a cassette encoding a ribozyme-flanked gRNA, which was successfully used in the Saccharomycotina yeasts *S. pastorianus* (Gorter de Vries et al., 2017), *Kluyveromyces* sp. and *Ogataea* sp. (Juergens et al., 2018). Cloning of specific gRNA cassettes targeting *SeMALT1, SeMALT2*/*T4*, and *SeMALT3* in pUDP002 resulted in pUDP062, pUDP063, and pUDP064, respectively. These plasmids were then transformed into *S. eubayanus* CBS 12357^T^, either alone or in combination with a 120-bp double stranded repair DNA fragment for the targeted Se*MALT* gene (Figure 1). In the absence of a repair fragment, transformation with a gRNA-expressing construct was expected to be fatal if both gene copies were cut, unless both breaks were repaired by non-homologous end joining (NHEJ) of the induced double strand breaks. However, transformation of *S. eubayanus* CBS 12357^T^ with pUDP062, pUDP063 or pUDP064 alone yielded 1100, 128, and 9 transformants, respectively. These numbers were not substantially different from those observed upon co-transformation of the corresponding repair fragments (3000, 114, and 13 colonies, respectively). Based on a set of 30 transformants, genome editing with the gRNA_*SeMALT*1_ yielded the lowest frequency of transformants in which both gene copies were deleted (3%). The *SeMALT3* gRNA performed better with a 7% frequency out of 13 transformants tested, while the gRNA targeting Se*MALT2/T4* showed an efficiency of 40% of accurate deletion of both copies of the two genes out of a set of eight transformants.

**Figure 4:**
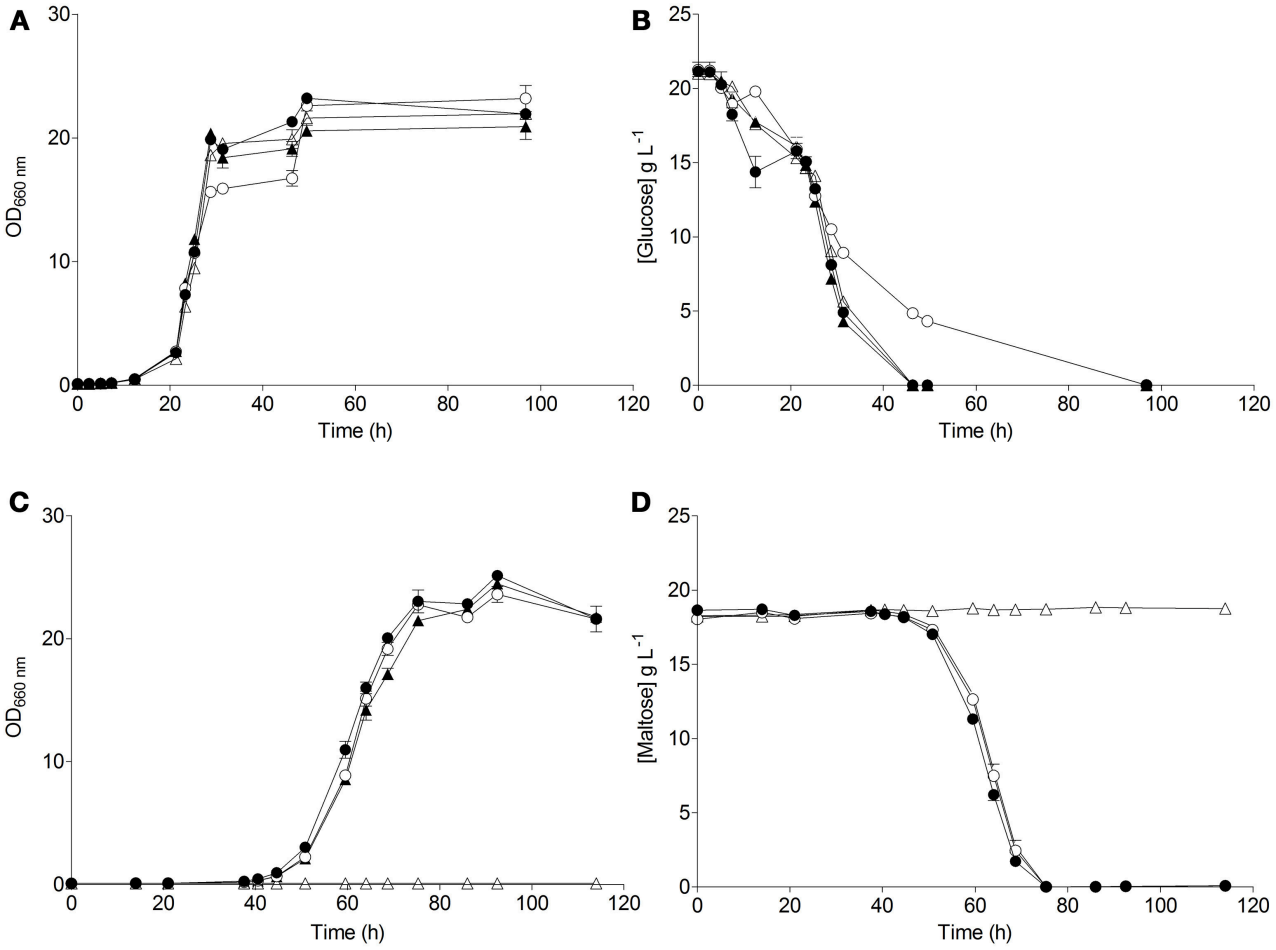
Characterization of the *S. eubayanus* strains (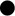) CBS 12357^T^, (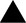) IMK816 (*SemalT1*Δ), (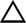) IMK817 (*SemalT2*Δ *SemalT4*Δ), and (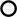) IMK818 (*SemalT3*Δ) during growth on glucose and maltose as sole carbon source. *S. eubayanus* strains were grown on SMG or SMM at 20°C. Growth on glucose **(A)** and on maltose **(C)** was monitored based on optical density measurement at 660 nm (OD_660nm_). Concentrations of glucose **(B)** and maltose **(D)** in culture supernatants were measured by HPLC. Data are presented as average and standard deviation of three biological replicates.

The resulting *S. eubayanus* deletion strains IMK816 (*SemalT1*Δ), IMK817 (*SemalT2*Δ *SemalT4*Δ), and IMK818 (*SemalT3*Δ), as well as the wild-type strain CBS 12357^T^, were grown in SMG and SMM media. While specific growth rates of all four strains in SMG were the same (0.22 h^−1^ at 20°C, Table 4), strain IMK187 (*SemalT2*Δ *SemalT4*Δ) did not grow on maltose (Figure 4). Conversely, strains IMK816 and IMK818 exhibited the same specific growth rate on maltose as the reference strain (0.17 h^−1^ at 20°C, Table 4). These data suggested that only *SeMALT2* and/or *SeMALT4* only contributed to growth on maltose of wild- type *S. eubayanus* CBS 12357^T^.

**Table 4:** Specific growth rates (h^−1^) of *S. eubayanus* CBS 12357^T^ (Libkind et al., 2011), *S. eubayanus* (*Se*) maltose transporter deletion mutants, *S. cerevisiae* (*Sc*) strains overexpressing individual *S. eubayanus* maltose transporter genes and the maltose-consumption-deficient host strain *S. cerevisiae* IMZ616 (Marques et al., 2017).

**Strain**	**Species**	**Relevant genotype or phenotype**	**Specific growth rate (h**^**−1**^**)**	
			**Glucose**	**Maltose**
CBS 12357^T^	*Se*	*SeMALT1 SeMALT2 SeMALT3 SeMALT4*	0.24 ± 0.003	0.17 ± 0.001
IMK816	*Se*	*SemalT1Δ* Se*MALT2 SeMALT3 SeMALT4*	0.22 ± 0.001	0.17 ± 0.000
IMK817	*Se*	*SeMALT1* Se*malT2Δ* Se*MALT3 SemalT4Δ*	0.22 ± 0.002	0.002 ± 0.000
IMK818	*Se*	*SeMALT1 SeMALT2 SemalT3Δ SeMALT4*	0.22 ± 0.001	0.17 ± 0.002
IMZ616	Sc	“*mal*Δ”	0.19 ± 0.002	0.00 ± 0.000
IMX1253	*Sc*	“*mal*Δ” *ScTEF1_*pr*_-SeMALT1-ScCYC1_*ter*_ ScMAL12*	0.20 ± 0.003	0.13 ± 0.002
IMX1254	*Sc*	“*mal*Δ” *ScTEF1_*pr*_-SeMALT2-ScCYC1_*ter*_ ScMAL12*	0.18 ± 0.002	0.13 ± 0.001
IMX1255	*Sc*	“*mal*Δ” *ScTEF1_*pr*_-SeMALT3-ScCYC1_*ter*_ ScMAL12*	0.19 ± 0.002	0.13 ± 0.004
IMX1365	*Sc*	“*mal*Δ” *ScTEF1_*pr*_-ScMAL11-ScCYC1_*ter*_ ScMAL12*	0.19 ± 0.001	0.099 ± 0.005

*S. eubayanus strains were grown on SMG and SMM media at 20°C. S. cerevisiae strains were grown on SM_U_G and SM_U_M at 20°C. Data represent average and standard deviation of three biological replicates. “malΔ” denotes the following genotype mal1Δ mal2Δ mal3Δ mph2Δ mph3Δ suc2Δ ima1Δ ima2Δ ima3Δ ima4Δ ima5Δ*.

### *SeMALT1, SeMALT2/T4*, and *SeMALT3* all support maltose uptake

For further functional analysis, *SeMALT1, SeMALT2*, and *SeMALT3* were expressed in a maltose-transporter negative *S. cerevisiae* strain background. *SeMALT4* was not included in this comparison, as it encodes a protein with the same amino acid sequence as *SeMALT2*. The maltose-negative *S. cerevisiae* strain IMZ616 originates from *S. cerevisiae* CEN.PK102-3A, which carries three *MAL loci* (*MAL1, MAL2*, and *MAL3*) (Basso et al., 2011). To eliminate growth on α-glucosides, these three *MAL* loci, *MPH2*, and *MPH3* as well as the α-glucoside hydrolase-encoding genes *SUC2* and *IMA1-5* were deleted, yielding *S. cerevisiae* IMK291 (Marques et al., 2017). Introduction of *cas9* into this strain yielded IMZ616 (Marques et al., 2018).

**Figure 5:**
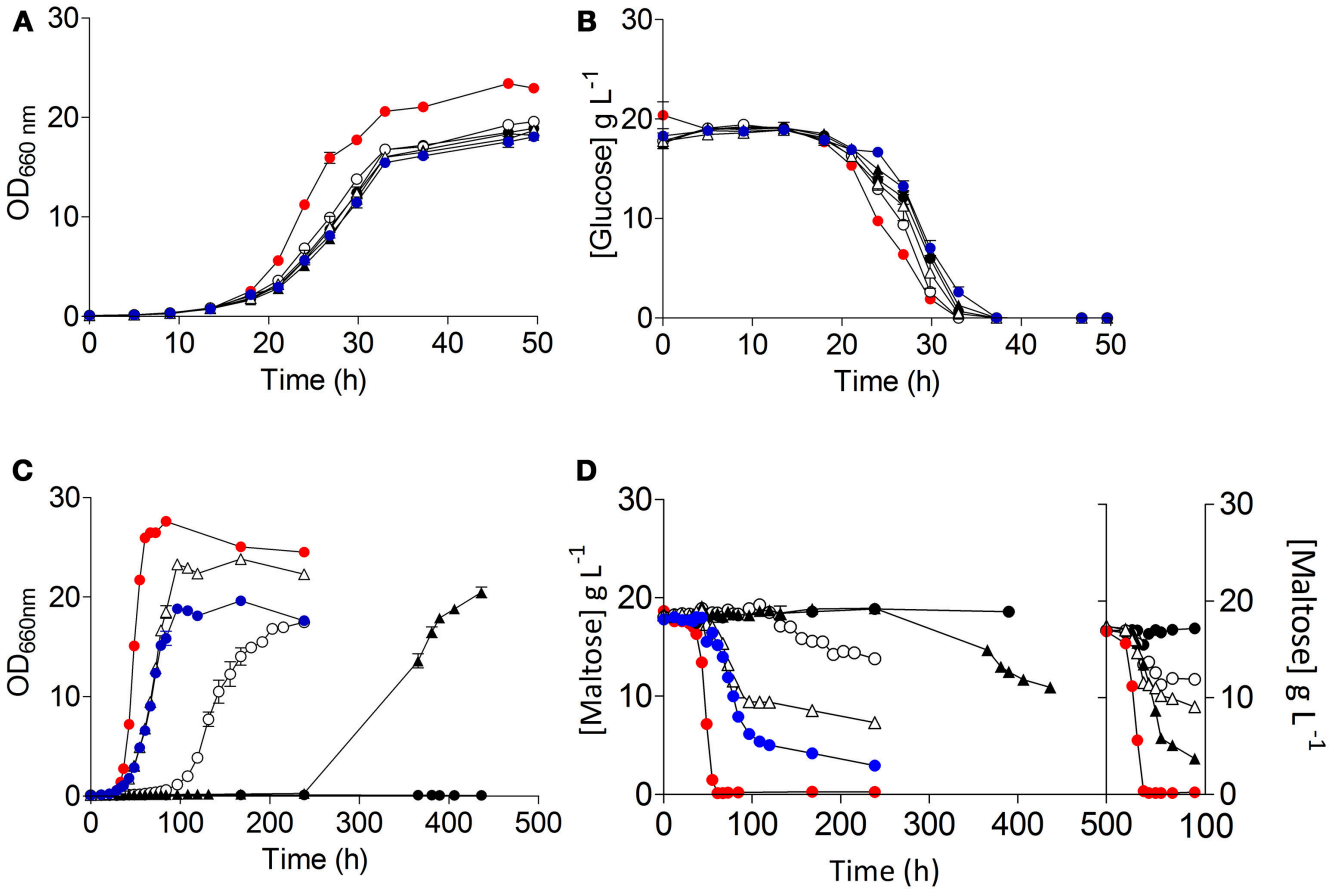
Characterization of the *S. cerevisiae* strains (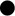) IMZ616 (Marques et al., 2018), (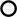) IMX1253 (Sc*TEF1*_*pr*_*-SeMALT1-ScCYC1*_*ter*_), (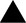) IMX1254 (Sc*TEF1*_*pr*_*-SeMALT2-ScCYC1*_*ter*_*)*, (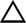) IMX1255 (Sc*TEF1*_*pr*_*-SeMALT3-ScCYC1*_*ter*_), (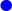) IMX1365 (*ScTEF1*_*pr*_-*ScMAL11-SeCYC1*_*ter*_), and (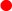) *S. eubayanus* CBS 12357^T^. The strains were grown in SM_U_G and SM_U_M at 20°C. Growth on glucose **(A)** and on maltose **(C)** was monitored based on optical density measurement at 660 nm (OD_660nm_). Concentrations of glucose **(B)** and maltose **(D)** in culture supernatants were measured by HPLC. **(D)** Shows the data of two consecutive batches. Data are presented as average and standard deviation of three biological replicates.

Restoration of growth on maltose of *S. cerevisiae* IMZ616 requires simultaneous expression of a maltose transporter and a maltase. Therefore, the three *S. eubayanus* transporter genes were cloned behind the constitutive *ScTEF1* promoter and, together with an expression cassette for the *ScMAL12* maltase gene, integrated at the *SGA1* locus of *S. cerevisiae* IMZ616 (Figure 2). The resulting *S. cerevisiae* strains IMX1253, IMX1254, IMX1255, which expressed *SeMALT1, SeMALT2*, and *SeMALT3*, respectively, were grown on SM_U_M. As expected, the host strain IMZ616 did not show any growth on maltose, while the three *SeMALT*-expressing strains showed different growth profiles on this disaccharide. Strain IMX1255 (*SeMALT3*) resumed growth after a lag phase of ~10 h and consumed half of the maltose supplied (Figure 5). Strains IMX1253 (*SeMALT1*) and IMX1254 (*SeMALT2*) showed lag phases of 100 and 250 h, respectively. However, after these lag phases, maltose was consumed. Strain IMX1254 (*SeMALT2*) consumed 75% of the supplied maltose in 150 h. In the same conditions the control strain IMX1365 co expressing *ScAGT1* and *ScMAL12* showed a short lag phase of 10 h and that was immediately followed by exponential growth, IMX1365 reached stationary phase and full maltose consumption in less than 100 h a performance comparable to the IMX1255 (*SeMALT3*) (Figure 5).

These delayed growth phenotypes of the Se*MALT*-expressing strains resemble those observed during growth on lactose of S. *cerevisiae* strains expressing the β-galactosidase (*LAC4*) and the lactose permease (*LAC12*) genes of *Kluyveromyces marxianus* and *Kluyveromyces lactis* (Domingues et al., 1999; Guimarães et al., 2008) In those studies, lactose utilization first had to be improved by laboratory evolution which resulted in lowering copy number of the plasmid harboring the permease and hydrolase genes as well as a short internal deletion located in the bi-directional promoter driving expression of the two genes (Guimarães et al., 2008). The same way, an adaptation step have been included in the workflow for complementation of the hexose transporter null (*hxt*^0^) strain EBYWV4000 with human glucose transporter (*GLUT*) (Boles and Oreb, 2018) suggesting that swapping transporter or implementing new assimilatory pathway remains non-trivial.

When, at the end of a first round of batch cultivation on maltose, cells of the *SeMALT*-expressing strains were transferred to fresh maltose medium, they all showed instantaneous growth at a specific growth rate of 0.13 h^−1^ (Table 4). Under the same conditions, *S. eubayanus* CBS 12357^T^ grew on maltose at a specific growth rate of 0.17 h^−1^ (Figure 5, Table 4). Even after transfer to fresh maltose medium, none of the heterologously expressed *SeMALT* transporters enabled full maltose consumption in these cultures. Similarly to the first cycle of batch cultivation on maltose, strain IMX1254 (*MALT2*) consumed 75% of the maltose supplied, while strains IMX1255 (Se*MALT3*) and IMX1253 (Se*MALT1*) consumed ca. 50 and 35 percentage even after prolonged incubation, none of the *S. cerevisiae* strains expressing an *SeMALT* gene nor *S. eubayanus* CBS 12357^T^ showed growth on maltotriose, while the positive control IMX1365 (*ScAGT1*) showed growth at a rate of 0.08 ± 0.007 h^−1^ (Figure S9).

In contrast to the deletion study, which suggested that only *Se*Malt2 and/or *Se*Malt4 were able to transport maltose, heterologous expression in the Mal^−^ *S. cerevisiae* strain IMZ616 showed that all four transporter genes encode transporters that, in combination with a maltase, allow growth on maltose.

### Slow growth of *SeMALT*-expressing *S. cerevisiae* strains is not caused by maltose accelerated death

**Figure 6:**
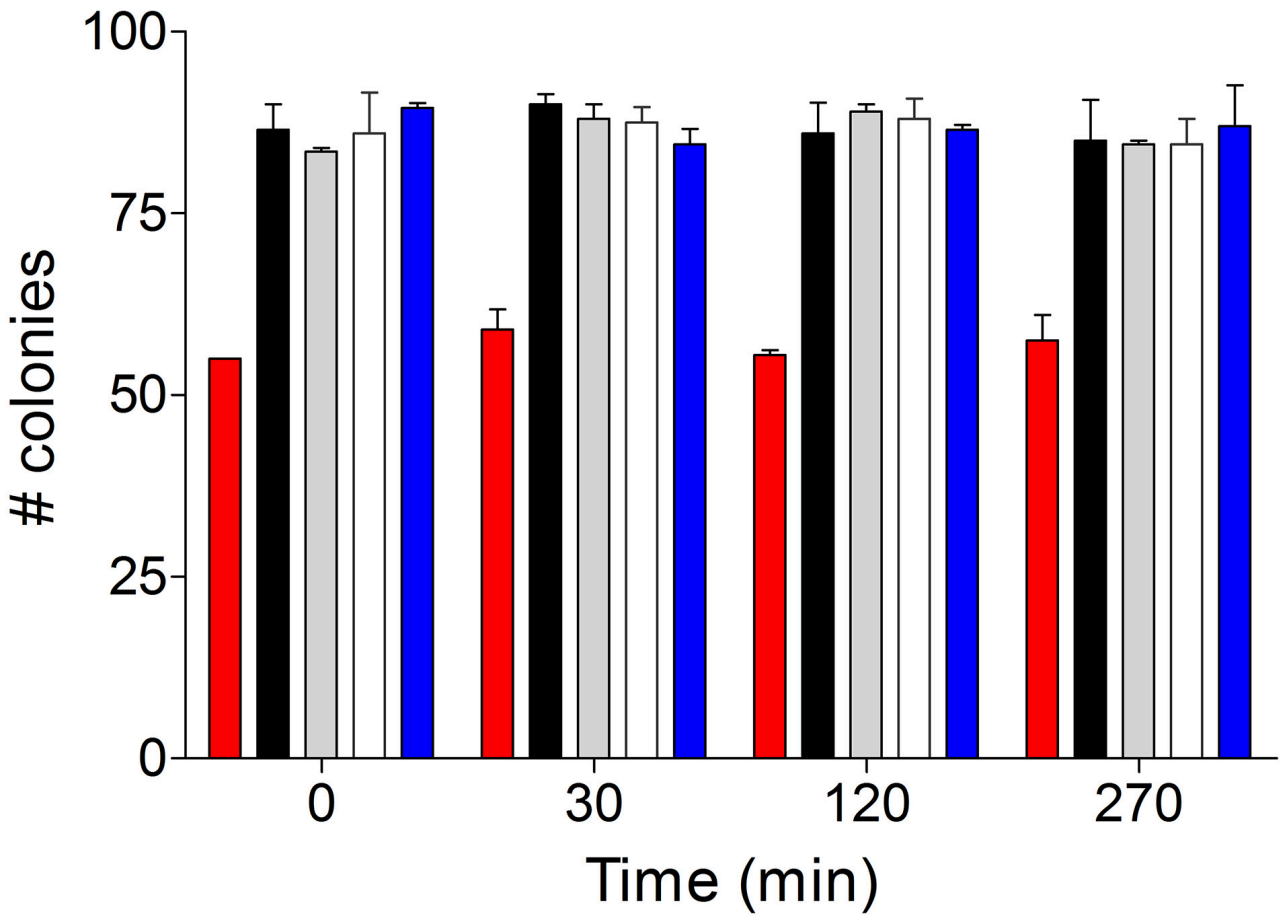
Cell viability after exposure to maltose of glucose-pregrown cultures of *S. eubayanus* CBS 12357^T^ (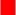), *S. cerevisiae* IMZ616 (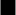), IMX1253 (*SeMALT1*) (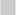), IMX1254 (*SeMALT2*) (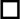) and IMX1255 (*SeMALT3*) (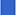). Cells from glucose-grown batch cultures were resuspended in SM. Prior addition of 20 g L^−1^ of maltose, the initial viability was measured by sorting 96 cells per strain on SM_U_G plates. The SM_U_G cultures were sampled after 30, 120 and 270 min. The viability data are represented as averages ± mean deviations of three independent experiments for each strain.

Since maltose is imported by proton symporters energized by the plasma-membrane proton-motive force (Serrano et al., 1986; van den Broek et al., 1994), an unrestricted influx of maltose can lead to a fast influx of protons (Jansen et al., 2004). Unless the resulting rate of proton influx can be countered by the proton-pumping plasma-membrane ATPase (Pma1; Serrano et al., 1986), dissipation of the proton motive force and cytosolic acidification can cause maltose-induced cell death. Indeed, pronounced maltose-accelerated death has been observed *S. cerevisiae* evolved an increased maltose-transport capacity (Jansen et al., 2004). To test whether this phenomenon was responsible for the observed delayed growth of *S. cerevisiae* strains expressing *S. eubayanus* maltose transporters, the corresponding *S. cerevisiae* strains (IMX1243, IMX1254, and IMX1255) were first grown on SMG. Upon reaching late exponential phase, cells after washing were transferred to SM medium (without C-source) to give an OD_660_ of 1.0. The resulting cell suspension was then sampled before and 30, 120, and 270 min after addition of 20 g.L^−1^maltose. From each sample, 96 cells were sorted using gated Forward scatter signal and side scatter signal intensities on SMG medium and the viability was estimated based on the number of growing colonies. Neither the three *SeMALT-*expressing *S. cerevisiae* strains, nor *S. eubayanus* CBS 12357^T^ or the Mal^−^ *S. cerevisiae* IMZ616 showed a decreased viability over a period of 270 min exposure to maltose (Figure 6). This result indicated that delayed growth of the *SeMALT*-expressing *S. cerevisiae* strains was not due to maltose accelerated death.

### In contrast to *SeMALT2/T4*, the transporter genes *SeMALT3* and *SeMALT1* were not efficiently transcribed in maltose grown cells

**Figure 7:**
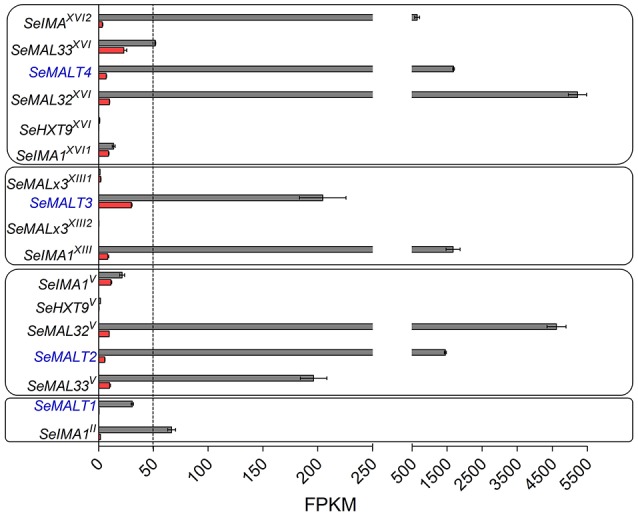
Expression of the maltose metabolism associated genes in *S. eubayanus* CBS 12357^T^. Transcript levels of maltose metabolism genes located at subtelomeric regions of CHRII, V, XII and XVI. FPKM gene expression values of *S. eubayanus* CBS 12357^T^ grown on SMG [glucose, (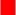)] and SMM [maltose, (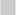)] at 20°C were calculated from duplicate RNA seq experiments (2 × 150 bp; 7.1 Gb) using the “Applying rpkm” method in the EdgeR package (Robinson et al., 2010). Data represent average and mean deviation of two biological replicates.

The inability of the double deletion mutant *S. eubayanus* IMK817 (Se*malT2*Δ-Se*malT4*Δ) to grow on maltose, despite the demonstration that *SeMALT1* and *SeMALT3* have the potential to encode functional maltose transporters, might indicate that *SeMALT1* and *SeMALT3* are not expressed in maltose-grown cultures. To investigate the impact of carbon sources on genome-wide transcript profiles and, more specifically, on transcriptional regulation of maltose metabolism genes, duplicate cultures of the *S. eubayanus* wild-type strain CBS 12357^T^ were grown on SMG and SMM and sampled in mid-exponential phase (OD_660nm_ = 12.5 ± 1.0). After mRNA isolation and processing, cDNA libraries were sequenced with Illumina sequencing technology. cDNA sequencing reads were mapped onto the newly annotated *S. eubayanus* CBS 12357^T^ genome assembly and used to calculate FPKM (fragments per kilobase of feature (gene) per million reads mapped) expression values. FPKM results represent normalized expression values that take into account gene length and sequencing depth. Statistical analysis showed that 125 genes were differentially expressed in the glucose- and maltose-grown cultures with a fold difference > 4 (Table S7). All four *S. eubayanus MALT* transporters were significantly upregulated during growth on maltose and three (*SeMALT2, SeMALT4*, and *SeMALT1*) were found among the ten most upregulated genes (Table 5). *SeMALT2* and *SeMALT4* exhibited 262- and 244-fold higher transcript levels during growth on maltose than during growth on glucose. *SeMALT1* and *SeMALT3* represented a substantially lower fold-difference between maltose- and glucose-grown cultures (67- and 6.8- fold, respectively; Figure 7, Table 5). The most pronounced difference between Se*MALT2/SeMALT4* and Se*MALT1/SeMALT3* concerned their expression level. The FPKM value of Se*MALT1* in maltose-grown cultures was 48-fold lower than that of *SeMALT2* and *SeMALT4* value (FPKM_*SeMALT*1_ = 30; FPKM_*SeMALT*2_ = 1,683, FPKM_*SeMALT*4_ = 1451). Similarly *SeMALT3* exhibited a FPKM value of only 200. In the same analysis, Se*ACT1* and the glycolytic gene Se*TDH3*, genes commonly used as internal standard in transcript analysis exhibited substrate-independent FPKM values of 1600 and 6000, respectively. The maltase genes that were physically associated to *SeMALT2* and *SeMALT4* (Figure 3) were also strongly overexpressed in maltose-grown cultures, representing the highest upregulation fold difference in expression in glucose- and maltose-grown cultures of *S. eubayanus* (Table 5). This result confirms the functionality of the bidirectional promoters controlling the maltase and transporter genes in the *S. eubayanus MAL* loci harboring *SeMALT2* and *SeMALT4*.

**Table 5:** Transcript level of the 15 most strongly upregulated genes in maltose-grown *S. eubayanus* CBS 12357^T^.

**CHR**	**Coordinates**	**Gene name**	**Description**	**FPKM**		**Fold-change**	**Adjusted *p*-value**
				**Glucose**	**Maltose**		
CHRXVI	13815–15572	*SeMAL32^*XVI*^*	Maltase (alpha-D-glucosidase)	9.9 ± 0.2	5218.4 ± 365	525.6	0
CHRV	569448–571205	*SeMAL32^*V*^*	Maltase (alpha-D-glucosidase)	9.6 ± 0.0	4620.4 ± 384	481.3	0
CHRV	566765-568606	*SeMALT2*	Maltose permease-member of the 12 TMB domain family of sugar transporters	5.5 ± 0.4	1451 ± 39	262.7	0
CHRXVI	16413–18254	*SeMALT4*	Maltose permease-member of the 12 TMB domain family of sugar transporters	6.9 ± 0.4	1683.8 ± 30	244.5	0
CHRIV	971754–973529	*SeDAK2*	Dihydroxyacetone kinase required for detoxification of dihydroxyacetone	19.2 ± 0.6	3342.3 ± 171	174.1	0
CHRII	8497–10338	*SeMALT1*	Maltose permease-member of the 12 TMB domain family of sugar transporters	0.5 ± 0.0	30.8 ± 1	67.7	3.2E−82
CHRXVI	25036–25626	*SeYJL218W*	Putative acetyltransferase	8.3 ± 2.3	528.3 ± 4	63.9	2.1E−246
CHRIV	968740–970695	*SeYFL054C*	Putative channel-like protein; similar to Fps1p	10.7 ± 2.2	529.6 ± 22	49.3	0
CHRII	5766–7535	*SeIMA1^*II*^*	Isomaltase, α−1,6-glucosidase; required for isomaltose utilization	1.6 ± 0.1	66.6 ± 5	41.7	2.7E−150
CHRIV	974654–975250	*SeREE1*	Cytoplasmic protein involved in the regulation of enolase	27.0 ± 1.1	1079.8 ± 76	40.0	2.3^E^-293
CHRV	564762–565970	*SeMAL33^*V*^*	MAL-activator protein	10.0 ± 1.2	196.3 ± 17	19.6	4.4E−171
CHRIV	512918–513952	*SeYRO2*	Protein with a putative role in response to acid stress	128.1 ± 6.9	2348.5 ± 110	18.3	2.5E−238
CHRXVI	732395–734113	*SeHXT13^*XVI*^*	Putative transmembrane polyol transporter	14.2 ± 3.9	202.2 ± 19	14.3	3.4E−113
CHRIV	552424–553068	*SeHSP26*	Small heat shock protein (sHSP) with chaperone activity	137.5 ± 19.5	1520.3 ± 33	11.1	1.2E−147
CHRVII	934199–934486	*SeSPG1*	Protein required for high temperature survival during stationary phase	6.3 ± 1.6	65.3 ± 5	10.3	2.3E−31

*FPKM gene expression values of S. eubayanus CBS 12357^T^ grown on SMG (glucose) and SMM (Maltose) were calculated from duplicate RNA seq experiments (2 × 150 bp; 7.1 Gb) using the “Applying rpkm” method from the EdgeR package (Robinson et al., 2010). p-values were calculated using DESeq and adjusted for multi-testing. The descriptions were based on similarity with S. cerevisiae orthologs*.

## Discussion

*S. eubayanus* is not only a key contributor to the hybrid genomes of current *S. pastorianus* lager brewing strain and a basis for developing new brewing yeast hybrids (Gibson et al., 2017) but is also directly used for brewing specialty lager beer (https://www.cnbc.com/2017/09/26/heineken-unveils-h41-a-beer-that-puts-yeast-into-focus.html) (Hittinger et al., 2018). This study provided an upgraded, near-complete genome sequence of the *S. eubayanus* type strain CBS 12357^T^and the first characterization of its four maltose transporter genes.

Complete and accurate *de novo* assembly of eukaryotic genomes has long been a major challenge. While short-read, high-coverage technologies such as the popular Illumina platforms enable accurate sequencing and assembly of unique genomic sequences, they cannot resolve repetitive sequences. As previously demonstrated for *S. cerevisiae* CEN.PK113-7D (Nijkamp et al., 2012; Salazar et al., 2017; Jenjaroenpun et al., 2018), use of the Oxford Nanopore MinION platform generated long-reads that enabled a near-complete assembly of 15 of the 16 chromosomes of *S. eubayanus* CBS 12357^T^ (Figure 3A). Correct assembly of subtelomeric regions, which are known for their high sequence redundancy, was especially important in the context of this study in view of the subtelomeric location of the four *MAL* loci in this strain (Figure 3B). Despite the massive sequencing coverage yielded by a single flow cell, which was invaluable for genome scaffold construction, the intrinsic higher error rate of MinION sequencing (Goodwin et al., 2015) required polishing with additional Illumina sequencing data. Although highly effective, as illustrated by the complete assembly of the four *MAL* loci and the use of resulting high-quality genome sequence information for transcriptome analysis, this approach could not correct all errors. In particular, INDELs causing omissions of single nucleotides in homopolymer regions were left in the final assembly, which is a known pitfall of the single molecule nanopore sequencing (Oxford Nanopore Technology MinIOn). Manual curation and, in particular, validation of relevant sequences by Sanger sequencing will be required to further refine the current genome sequence of *S. eubayanus* CBS 12357^T^.

Also the first application of CRISPR-Cas9-assisted genome editing in *S. eubayanus* reported in this study was greatly facilitated by the availability of an accurate genome sequence. Resolution of the sequences of the four *SeMALT* genes enabled optimization of the gRNA spacer selection, thereby minimizing the risk of undesirable off-target events. Although targeting efficiencies of the employed ribozyme-flanked-gRNA expression system (Gorter de Vries et al., 2017), which ranged from 3 to 40% were not ideal, Cas9-assisted gene deletion offered clear advantages over traditional methods that rely on a double crossover event that inserts a DNA fragment containing a selection marker in the recipient strain's genome (Baudin et al., 1993; Wilson et al., 2000). Cas9-assisted genome editing in S*. eubayanus* did not make use of a marker cassette and, most importantly, enabled simultaneous marker-free editing both alleles of the *SeMALT1* or the *SeMALT3* gene. In the case of *SeMALT2* and *SeMALT4*, a single transformation was even sufficient to delete both alleles of two genes (DiCarlo et al., 2013; Mans et al., 2015). Achieving these objectives with conventional techniques would have been extremely time consuming as multiple rounds of transformation and marker recovery would be required. Depending on consumer acceptance and regulations in place (Hall, 2016; Waltz, 2016), marker-free, Cas9-assisted genome editing of *S. eubayanus* may be combined with the generation of new *Saccharomyces* hybrids to accelerate development to novel brewing strains.

Cas9-mediated gene disruption and transcriptome analysis in *S. eubayanus* CBS 12357 showed that, in maltose-grown cultures, *SeMALT2* and *SeMALT4* were predominantly responsible for maltose uptake. These transporters are located within two nearly identical (97% identity) *SeMAL* loci that strongly resemble canonical *S. cerevisiae MAL* loci (Charron et al., 1989; Chow et al., 1989; Vidgren et al., 2005). Although two other genes, Se*MALT3* and Se*MALT1*, could restore growth upon their expression in the Mal^−^ *S. cerevisiae* strain IMZ616, their low expression levels in *S. eubayanus* CBS 12357^T^ were apparently not sufficient to support growth on maltose when *SeMALT2* and *SeMALT4* were both deleted (Figures 3, 7). This study did not provide new insights into the origin of *MTT1/MTY1* (Dietvorst et al., 2005; Salema-Oom et al., 2005) and *SeAGT1* (Nakao et al., 2009; Vidgren and Londesborough, 2012; Hebly et al., 2015) in industrial *S. pastorianus* strains as, consistent with earlier observations (Baker et al., 2015), no genes with strong sequence similarity to these maltose transporter genes were identified in the improved genome sequence of *S. eubayanus* CBS 12357^T^.

The low expression levels of *SeMALT1* and *SeMALT3* may be related to their genomic context, as they were located in atypical Se*MAL* loci on CHRII and CHRXIII, respectively (Figure 3B). Assuming that these two atypical Se*MAL* loci evolved from a complete *MAL* locus, loss of the maltase gene may have disrupted the bi-directional promoter that, in *S. cerevisiae*, controls expression of both the maltose and the maltose transporter genes (Levine et al., 1992). In *S. cerevisiae*, the maltose regulator Mal63 binds two regulatory sites (5′MGSN_9_MGS3′) located between positions −465 and −579 in the region separating the two divergent genes (Sirenko et al., 1995). While two of these elements were also found in the promoter regions of *SeMALT2* and *SeMALT4* (Figure S8), the *SeMALT1* and *SeMALT3* promoters each harbored only a single element (Figure S8). Alternatively, low expression of *SeMALT1* and *SeMALT3* in maltose-grown cultures may reflect a sub-functionalization that, during evolution, led to a different regulation and/or catalytic properties of the encoded transporters (Ohno, 1970; Hughes, 1994). Indeed, such a sub-functionalization has been experimentally reconstructed for yeast α-glucoside hydrolases (Voordeckers et al., 2012).

None of the four Se*MALT* genes identified in *S. eubayanus* CBS 12357^T^ were found to encode a functional maltotriose transporter. Although, the *S. eubayanus* Patagonian lineage is unlikely to have contributed the *S. eubayanus* subgenome of *S. pastorianus* lager brewing strains (Nakao et al., 2009), this observation would be consistent with the notion that *S. cerevisiae* has contributed the vital ability to ferment this trisaccharide, which is abundantly present in wort as in contrast multiple *S. cerevisiae* ale strains mainly issued from the beer 1 and 2 groups have been shown to use this sugar (Gallone et al., 2016). However, CBS 12357 is a representative of sole the Patagonia B group, one of five groups defined based of the phylogenetic distribution of *S. eubayanus* strains isolated so far (Peris et al., 2016). Therefore, we cannot at all exclude the possibility that this brewing relevant phenotypic trait of lager yeast would originate from the *S. eubayanus* parent. The only elements so far that could tilt toward this hypothesis are very fragmented sequencing data of an isolate from the Holartic group, that suggested occurrence of *S. eubayanus* ortholog of the *S. cerevisiae AGT1/MAL11* gene (Hebly et al., 2015). Therefore, the resource and methodology used in this study paved the way for further exploration of the diversity of *S. eubayanus* population and elucidation of *S. eubayanus* parental lineage of *S. pastorianus*.

## Author contributions

J-MD, JP, AB, NB designed experiments. AB, NB, JG performed experiments. MB, NB, and J-MD performed bioinformatics work. J-MD, JP, AB, NB, JG analyzed result data. J-MD and JP wrote the manuscript. All authors read and approved the final manuscript.

### Conflict of interest statement

The authors declare that the research was conducted in the absence of any commercial or financial relationships that could be construed as a potential conflict of interest.
