# Supplementary material for: Structural, Physiological and Regulatory Analysis of Maltose Transporter Genes in Saccharomyces eubayanus CBS 12357T
Source: Front Microbiol. 2018 Aug 10;9:1786. doi: 10.3389/fmicb.2018.01786 (PMC6097016; doi:10.3389/fmicb.2018.01786)
Supplement: Supplementary file 1 [file Data_Sheet_1.docx]

# Supplemental Materials for Structural, physiological and regulatory analysis of maltose transporter genes in *Saccharomyces eubayanus* CBS 12357^T^.

Anja Brickwedde^#^, Nick Brouwers^#^, Marcel van den Broek, Joan S. Gallego Murillo, Julie Fraiture, Jack T. Pronk and Jean-Marc G. Daran*

* Corresponding author: Department of Biotechnology, Delft University of Technology, van der Maasweg 9, 2629 HZ Delft, The Netherlands

**Table S5:** Primers used in this study

| Primer | Sequence | Purpose |
| --- | --- | --- |
| 7812 | TCATGTAATTAGTTATGTCACGCTTACATTC | Amplification of p426-TEF-amdS backbone for Gibson Assembly |
| 5921 | AAAACTTAGATTAGATTGCTATGCTTTCTTTCTAATGAGC | Amplification of p426-TEF-amdS backbone for Gibson Assembly |
| 10491 | GCTCATTAGAAAGAAAGCATAGCAATCTAATCTAAGTTTTAAAGTTTCGGTATACTTAGCAGACAG | Amplification of Se*MALT1* from *S. eubayanus* CBS 12357 for Gibson Assembly |
| 10492 | GGAGGGCGTGAATGTAAGCGTGACATAACTAATTACATGATACCCTAATCAAGTAAATAGATAATAAAGTTAATGTG | Amplification of Se*MALT1* from *S. eubayanus* CBS 12357 for Gibson Assembly |
| 10632 | GGAGGGCGTGAATGTAAGCGTGACATAACTAATTACATGATGCGCTAAGAGTCATCAAT | Amplification of Se*MALT2* from *S. eubayanus* CBS 12357 for Gibson Assembly |
| 10633 | GCTCATTAGAAAGAAAGCATAGCAATCTAATCTAAGTTTTGAGGCGTGATATGCTCCAT | Amplification of Se*MALT2* from *S. eubayanus* CBS 12357 for Gibson Assembly |
| 10671 | GGAGGGCGTGAATGTAAGCGTGACATAACTAATTACATGATGTCAGATAACAAAACCAGATACC | Amplification of Se*MALT3* from *S. eubayanus* CBS 12357 for Gibson Assembly |
| 10672 | GCTCATTAGAAAGAAAGCATAGCAATCTAATCTAAGTTTTCGATAGAATATCCTGCTGAACC | Amplification of Se*MALT3* from *S. eubayanus* CBS 12357 for Gibson Assembly |
| 9940 | GCTCATTAGAAAGAAAGCATAGCAATCTAATCTAAGTTTTCAACGTACCGGGCTTGAGGGACATACAG | Amplification of *ScAGT1* from *S. cerevisiae*  CEN.PK113-7D |
| 9941 | GGAGGGCGTGAATGTAAGCGTGACATAACTAATTACATGACTAGCTGAGGGTTTTGGGAGCAGTCAAAGG | Amplification of *ScAGT1* from *S. cerevisiae*  CEN.PK113-7D |
| 10178 | GGGAACAAAAGCTGGAGCTCATAG | Out-Out diagnostic PCR of Gibson Assembly constructs |
| 9947 | TACCGGCCGCAAATTAAAGC | Out-Out diagnostic PCR of Gibson Assembly constructs |
| 10627 | TTATAGGCGGGATGGGATGTTC | In-Out diagnostic PCR of Se*MALT1* Gibson Assembly construct |
| 10628 | CTGGCCTTCCAGACCGTTATAC | In-Out diagnostic PCR of Se*MALT2/4* Gibson Assembly construct |
| 10629 | TCTTCTAATGGTGGTCGCCTTC | In-Out diagnostic PCR of Se*MALT3* Gibson Assembly construct |
| 9036 | TTTACAATATAGTGATAATCGTGGACTAGAGCAAGATTTCAAATAAGTAACAGCAGCAAACATAGCTTCAAAATGTTTCTACTCCTTTTTTAC | Amplification of transporter gene expression cassette for construction of strains with transporter and Sc*MAL12* |
| 9039 | CACCTTTCGAGAGGACGATGCCCGTGTCTAAATGATTCGACCAGCCTAAGAATGTTCAACGCCGCAAATTAAAGCCTTCG | Amplification of transporter gene expression cassette for construction of strains with transporter and Sc*MAL12* |
| 9596 | TGTAAATATCTAGGAAATACACTTGTGTATACTTCTCGCTTTTCTTTTATTTTTTTTTGTAGTTTATCATTATCAATACTCGCCATTTC | Amplification of Sc*MAL12* expression cassette for construction of strains with transporter and Sc*MAL12* |
| 9355 | TGTAAATATCTAGGAAATACACTTGTGTATACTTCTCGCTTTTCTTTTATTTTTTTTTGTAGTTTATCATTATCAATACTCGCCATTTC | Amplification of Sc*MAL12* expression cassette for construction of strains with transporter and Sc*MAL12* |
| 5095 | GTGAGGTAAGTGTGCAAAGG | Out-Out diagnostic PCR of constructed guide RNA plasmids |
| 7219 | GTCTAACTCCTTCCTTTTCGG | Out-Out diagnostic PCR of constructed guide RNA plasmids |
| 4226 | ACTCGTACAAGGTGCTTTTAACTTG | Out-Out diagnostic PCR of integration in IMZ616 sga1 |
| 4224 | TTGATGTAAATATCTAGGAAATACACTTG | Out-Out diagnostic PCR of integration in IMZ616 sga1 |
| 11850 | GAAGGGTCTATCTTCAATATTGAATAGAAAGAGAAACGAAAGCGATTCGATTTCCAGTAATTTAGCCTAGGTACTCTATGTTGGGCCATCATTGATTTACCCGAAACTGCCGGTAGAACT | *SeMALT1* repair (+) Deletion +63 -+1600 |
| 11851 | AGTTCTACCGGCAGTTTCGGGTAAATCAATGATGGCCCAACATAGAGTACCTAGGCTAAATTACTGGAAATCGAATCGCTTTCGTTTCTCTTTCTATTCAATATTGAAGATAGACCCTTC | *SeMALT1* repair (-) Deletion +63 -+1600 |
| 11328 | ATGAAGGGTCTATCCTCAATGATAAATAGAAAGAAGTGCAACGGTAACTCGAGCTCAATAGAAGATATGAAAGCTTCCGCCGAGGAGAGAGAGCAAAGCACCCCATCTCTAATGGATTGA | *SeMALT2/T4* repair (+) Deletion +60 - +1782 |
| 11329 | TCAATCCATTAGAGATGGGGTGCTTTGCTCTCTCTCCTCGGCGGAAGCTTTCATATCTTCTATTGAGCTCGAGTTACCGTTGCACTTCTTTCTATTTATCATTGAGGATAGACCCTTCAT | *SeMALT2/T4* repair (-) Deletion +60 - +1782 |
| 11330 | ATGAAGGGCTTATCCTCACTGATAAACAGAAAAAAAAACAAGATTGACTCTAATTCAAATGATATGGAAACTTCCATGGTGGAAGAAGGGCGAAGCACACCATCTATTACGAATTTATGA | *SeMALT3* repair (+) Deletion +60 - +1785 |
| 11331 | TCATAAATTCGTAATAGATGGTGTGCTTCGCCCTTCTTCCACCATGGAAGTTTCCATATCATTTGAATTAGAGTCAATCTTGTTTTTTTTTCTGTTTATCAGTGAGGATAAGCCCTTCAT | *SeMALT3* repair (-) Deletion +60 - +1785 |
| 11671 | AGGTTCCTGGGCAGTGAAGC | *SeMALT1* deletion check |
| 11672 | AGGTCCAAGTCCTCTGTAAG | *SeMALT1* deletion check |
| 11673 | CCCTGAATGATCTGGTGAAC | *SeMALT*2/T4 deletion check |
| 11674 | AACACCCGCTATATTCCTCG | *SeMALT*2/T4 deletion check |
| 11675 | CATAGCTGGTACAGGATACG | *SeMALT3* deletion check |
| 11676 | GCCGTGAATAGCTTAAGGTG | *SeMALT3* deletion check |
| 5043 | CGAGCAAATGCCTGCAAATCG | Transporter integration check Out-In |
| 942 | AGCAGCAAACAGCGTCTTGTC | Transporter integration check In-Out |

**Table S6: List of genes added or modified in the new *S. eubayanus* CBS 12357T reference genome and annotations.** The descriptions were based on similarity with *S. cerevisiae* orthologs.

|  |  |  |  | Similarity with *S. cerevisiae* gene | | |
| --- | --- | --- | --- | --- | --- | --- |
|  |  |  |  | Systematic |  |  |
| CHR | Start | End | Length | Name | Name | Description |
| *Se*CHRI | 5572 | 6660 | 1089 | YJR153W | *PGU1* | Endo-polygalacturonase |
| *Se*CHRI | 9928 | 14679 | 4752 | YAL063C | *FLO9* | Flo1p homolog |
| *Se*CHRI | 10391 | 13801 | 3411 | YAL064W-B | YAL064W-B | Fungal-specific protein of unknown function |
| *Se*CHRI | 177093 | 179285 | 2193 | YAR035W | *YAT1* | Outer carnitine acetyltransferase, mitochondrial |
| *Se*CHRI | 187599 | 193115 | 5517 | YAR050W | *FLO1* | FLO1 putative cell wall glycoprotein |
| *Se*CHRI | 194550 | 195545 | 996 | YJL213W | YJL213W | Similarity to *Methanobacterium* aryldialkylphosphatase related protein |
| *Se*CHRI | 197031 | 197744 | 714 | YDR533C | *HSP31* | Strong similarity to hypothetical proteins YPL280w, YOR391c and YMR322c |
| *Se*CHRI | 199000 | 199770 | 771 | YKL071W | YKL071W | Weak similarity to *A. parasiticus* nor-1 protein |
| *Se*CHRI | 202624 | 204504 | 1881 | YIL169C | YIL169C | Similarity to glucan 1,4-alpha-glucosidase Sta1p and YAR066w |
| *Se*CHRII | 10694 | 11368 | 675 | YFL060C | *SNO3* | Proximal ORF, stationary phase induced gene |
| *Se*CHRII | 11798 | 12739 | 942 | YFL059W | *SNZ3* | Snooze: stationary phase-induced gene family |
| *Se*CHRII | 13335 | 14357 | 1023 | YDL244W | *THI13* | Thiamine biosynthetic enzyme |
| *Se*CHRII | 187048 | 194272 | 7224 | YBL017C | *PEP1* | Carboxypeptidase Y sorting receptor in late Golgi |
| *Se*CHRII | 204314 | 205498 | 1185 | YBL013W | *FMT1* | Probable met-tRNA formyltransferase, mitochondrial |
| *Se*CHRII | 553563 | 564431 | 10869 | YDR150W | *NUM1* | Protein with variable number of tandem repeats of a 64 amino-acid polypeptide |
| *Se*CHRII | 870964 | 871440 | 477 | YDR319C | *YFT2* | Member of the highly conserved FIT family of proteins involved in triglyceride droplet biosynthesis |
| *Se*CHRII | 920553 | 922265 | 1713 | YDR342C | *HXT7* | Hexose transporter |
| *Se*CHRII | 925500 | 930623 | 5124 | YDR345C | *HXT3* | Low-affinity glucose transporter |
| *Se*CHRII | 1067774 | 1072780 | 5007 | YDR420W | *HKR1* | Type 1 membrane protein with EF hand motif |
| *Se*CHRIII | 264091 | 270210 | 6120 | YCR089W | *FIG2* | predicted GPI-anchored cell wall protein |
| *Se*CHRIV | 38153 | 39994 | 1842 | YDL229W | *SSB1* | Heat shock protein of HSP70 family |
| *Se*CHRIV | 116188 | 117552 | 1365 | YDL189W | *RBS1* | Hypothetical protein |
| *Se*CHRIV | 117842 | 118978 | 1137 | YDL188C | *PPH22* | Serine-threonine protein phosphatase 2A |
| *Se*CHRIV | 220021 | 221343 | 1323 | YDL131W | *LYS21* | Homocitrate synthase, highly homologous to YDL182W |
| *Se*CHRIV | 373695 | 377804 | 4110 | YDL037C | *BSC1* | Strong similarity to glucan 1,4-alpha-glucosidase |
| *Se*CHRIV | 599827 | 601230 | 1404 | YBR092C | *PHO3* | Acid phosphatase, constitutive |
| *Se*CHRIV | 601685 | 603088 | 1404 | YBR092C | *PHO3* | Acid phosphatase, constitutive |
| *Se*CHRIV | 853943 | 855004 | 1062 | YBR231C | *SWC5* | Similarity to human p97 homologous protein |
| *Se*CHRV | 26219 | 27724 | 1506 | YNR073C | YNR073C | Strong similarity to *E.coli* D-mannonate oxidoreductase, identical to YEL070w |
| *Se*CHRV | 28195 | 29916 | 1722 | YEL069C | *HXT13* | Hexose transporter |
| *Se*CHRV | 195052 | 198240 | 3189 | YER017C | *AFG3* | ATP-dependent metalloprotease |
| *Se*CHRV | 577191 | 577442 | 252 | YHL047C | *ARN2* | Similarity to *C. carbonum* toxin pump |
| *Se*CHRVI | 3434 | 6913 | 3480 | YAL063C | *FLO9* | Flo1p homolog |
| *Se*CHRVI | 192972 | 193928 | 957 | YFR034C | *PHO4* | Myc-type helix-loop-helix transcription factor |
| *Se*CHRVI | 228124 | 238437 | 10314 | YDR134C | YDR134C | Strong similarity to Flo1p |
| *Se*CHRVI | 256654 | 257742 | 1089 | YJR153W | *PGU1* | Endo-polygalacturonase |
| *Se*CHRVII | 6946 | 8559 | 1614 | YIR039C | YPS6 | GPI-anchored aspartic protease |
| *Se*CHRVII | 606893 | 607519 | 627 | YPR071W | YPR071W | Strong similarity to YIL029c |
| *Se*CHRVIII | 26976 | 29960 | 2985 | YDR534C | *FIT1* | Similarity to YOR383c,Sta1p and pig mucin |
| *Se*CHRVIII | 253415 | 254362 | 948 | YOR083W | *WHI5* | Weak similarity to YKR091w |
| *Se*CHRIX | 4707 | 5837 | 1131 | YNL134C | YNL134C | Similarity to C. carbonum *toxD* gene |
| *Se*CHRIX | 6568 | 8187 | 1620 | YIR039C | *YPS6* | GPI-anchored aspartic protease |
| *Se*CHRIX | 8612 | 9748 | 1137 | YFL062W | *COS4* | Protein with strong similarity to subtelomerically-encoded proteins s |
| *Se*CHRIX | 29310 | 30941 | 1632 | YIL159W | *BNR1* | Bni1p-related protein, helps regulate reorganization of the actin cytoskeleton |
| *Se*CHRIX | 238441 | 239202 | 762 | YIL049W | *DFG10* | Protein involved in filamentous growth, cell polarity, and cellular elongation |
| *Se*CHRIX | 365797 | 369945 | 4149 | YIR019C | *MUC1* | Cell surface flocculin with structure similar to serine threonine-rich GPI-anchored cell wall proteins |
| *Se*CHRIX | 371169 | 371381 | 213 | YIR020W-A | YIR020W-A | Dubious open reading frame |
| *Se*CHRX | 3742 | 5622 | 1881 | YIL169C | YIL169C | Similarity to glucan 1,4-alpha-glucosidase Sta1p and YAR066w |
| *Se*CHRX | 8474 | 9253 | 780 | YKL071W | YKL071W | Weak similarity to *A. parasiticus* nor-1 protein |
| *Se*CHRX | 10614 | 11939 | 1326 | YJL213W | YJL213W | Similarity to *Methanobacterium* aryldialkylphosphatase related protein |
| *Se*CHRX | 13383 | 17423 | 4041 | YAL063C | *FLO9* | Flo1p homolog |
| *Se*CHRX | 23126 | 24451 | 1326 | YJL213W | YJL213W | Similarity to *Methanobacterium* aryldialkylphosphatase related protein |
| *Se*CHRX | 102189 | 103517 | 1329 | YJL164C | *TPK1* | Putative catalytic subunit of cAMP-dependent protein kinase |
| *Se*CHRX | 145708 | 146898 | 1191 | YKR059W | *TIF1* | Translation initiation factor eIF4A |
| *Se*CHRX | 287825 | 288391 | 567 | YJL075C | *APQ13* | questionable ORF |
| *Se*CHRX | 348364 | 349281 | 918 | YJL043W | YJL043W | Similarity to hypothetical protein YKR015c |
| *Se*CHRX | 432474 | 434615 | 2142 | YJR004C | *SAG1* | Alpha-agglutinin |
| *Se*CHRX | 702344 | 705556 | 3213 | YJR150C | *DAN1* | Protein induced during anaerobic growth |
| *Se*CHRX | 732395 | 734113 | 1719 | YEL069C | *HXT13* | Hexose transporter |
| *Se*CHRX | 734531 | 736036 | 1506 | YNR073C | YNR073C | Strong similarity to *E. coli* D-mannonate oxidoreductase, identical to YEL070w |
| *Se*CHRX | 743018 | 744151 | 1134 | YFL062W | *COS4* | Protein with strong similarity to subtelomerically-encoded proteins |
| *Se*CHRX | 744577 | 746190 | 1614 | YIR039C | *YPS6* | GPI-anchored aspartic protease |
| *Se*CHRXI | 54859 | 56775 | 1917 | YKL198C | *PTK1* | Probable serine threonine-specific protein kinase (EC 2.7.1.-) |
| *Se*CHRXI | 545410 | 545940 | 531 | YKR062W | *TFA2* | Small subunit of TFIIE transcription factor |
| *Se*CHRXI | 635126 | 639253 | 4128 | YKR102W | *FLO10* | Protein with similarity to flocculation protein Flo1p |
| *Se*CHRXII | 29343 | 32663 | 3321 | YAL063C | *FLO9* | Putative Flo1p homolog |
| *Se*CHRXII | 463503 | 464000 | 498 | YLR154C-H | YLR154C | Hypothetical protein |
| *Se*CHRXII | 519195 | 527990 | 8796 | YJL084C | *ART3* | Similarity to hypothetical protein YKR021w |
| *Se*CHRXII | 561132 | 570266 | 9135 | YJL084C | *ART3* | Similarity to hypothetical protein YKR021w |
| *Se*CHRXII | 644658 | 644999 | 342 | YLR204W | *QRI5* | Protein of unknown function |
| *Se*CHRXII | 661072 | 663138 | 2067 | YLR214W | *FRE1* | Ferric (and cupric) reductase |
| *Se*CHRXII | 664035 | 666101 | 2067 | YLR214W | *FRE1* | Ferric (and cupric) reductase |
| *Se*CHRXII | 1089672 | 1090070 | 399 | YLR437C | *DIF1* | Protein that regulates nuclear localization of Rnr2p and Rnr4p |
| *Se*CHRXIII | 277902 | 278318 | 417 | YMR003W | *AIM34* | Hypothetical protein |
| *Se*CHRXIII | 601432 | 603129 | 1698 | YMR173W | *DDR48* | Flocculent specific protein |
| *Se*CHRXIII | 904661 | 907945 | 3285 | YMR317W | YMR317W | Similarity to mucins, glucan 1,4-alpha-glucosidase and exo-alpha-sialidase |
| *Se*CHRXIII | 916196 | 917173 | 978 | YPL273W | *SAM4* | Strong similarity to hypothetical protein YLL062c |
| *Se*CHRXIII | 917562 | 919325 | 1764 | YPL274W | *SAM3* | Strong similarity to amino-acid transport proteins |
| *Se*CHRXIII | 923767 | 925488 | 1722 | YEL069C | *HXT13* | Hexose transporter |
| *Se*CHRXIII | 925958 | 927463 | 1506 | YNR073C | YNR073C | Strong similarity to *E. coli* D-mannonate oxidoreductase, identical to YEL070w |
| *Se*CHRXIII | 928789 | 929463 | 675 | YFL060C | *SNO3* | *SNZ2* proximal ORF, stationary phase induced gene |
| *Se*CHRXIII | 929892 | 930788 | 897 | YFL059W | *SNZ3* | Snooze: stationary phase-induced gene family |
| *Se*CHRXIII | 931430 | 932452 | 1023 | YDL244W | *THI13* | Thiamine biosynthetic enzyme |
| *Se*CHRXIV | 19114 | 22533 | 3420 | YNL327W | *EGT2* | Cell-cycle regulation protein |
| *Se*CHRXIV | 128069 | 129781 | 1713 | YNL270C | *ALP1* | Protein highly homologous to permeases Can1p and Lyp1p for basic amino acids |
| *Se*CHRXIV | 130734 | 132569 | 1836 | YNL268W | *LYP1* | Lysine permease |
| *Se*CHRXIV | 190720 | 192210 | 1491 | YNL240C | *NAR1* | Strong similarity to *K. marxianus* LET1 protein |
| *Se*CHRXIV | 549110 | 556552 | 7443 | YBL017C | *PEP1* | Carboxypeptidase Y sorting receptor in late Golgi |
| *Se*CHRXIV | 641192 | 642412 | 1221 | YNR012W | *URK1* | Uridine kinase |
| *Se*CHRXIV | 697011 | 699341 | 2331 | YNR044W | *AGA1* | Anchorage subunit of a-agglutinin |
| *Se*CHRXV | 732884 | 734602 | 1719 | YEL069C | *HXT13* | Hexose transporter |
| *Se*CHRXV | 735045 | 736550 | 1506 | YNR073C | YNR073C | Strong similarity to E.coli D-mannonate oxidoreductase, identical to YEL070w |
| *Se*CHRXVI | 2599 | 2961 | 363 | YOL161C | *PAU20* | Strong similarity to members of the Srp1p/Tip1p family |
| *Se*CHRXVI | 4738 | 6558 | 1821 | YOL158C | *ENB1* | Similarity to subtelomeric encoded proteins |
| *Se*CHRXVI | 7603 | 7851 | 249 | YHL047C | *ARN2* | Similarity to *C. carbonum* toxin pump |
| *Se*CHRXVI | 7895 | 9664 | 1770 | YGR287C | *IMA1* | Strong similarity to maltase |
| *Se*CHRXVI | 11123 | 12841 | 1719 | YJL219W | *HXT9* | High-affinity hexose transporter |
| *Se*CHRXVI | 13815 | 15572 | 1758 | YBR299W | *MAL32* | Maltase (EC 3.2.1.20) |
| *Se*CHRXVI | 16413 | 18254 | 1842 | YBR298C | *MAL31* | Maltose permease |
| *Se*CHRXVI | 21820 | 23589 | 1770 | YGR287C | *IMA1* | Strong similarity to maltase |
| *Se*CHRXVI | 907607 | 909334 | 1728 | YKR105C | *VBA5* | Strong similarity to Sge1p and hypothetical protein YCL069w |

**Table S7: Genes with a │fold-difference│in expression level ≥ 4 in glucose and maltose-grown cultures of *S. eubayanus* CBS 12357^T^.** Data represent average expression intensity and mean deviation of two replicate genome-wide transcript datasets.

|  |  |  | Similarity to *S.cerevisiae* gene | |  |  |  |  |  |
| --- | --- | --- | --- | --- | --- | --- | --- | --- | --- |
|  |  |  |  |  |  | Glucose | Maltose | Fold | Adjusted |
|  | start | stop | Name | Syst name | description | FPKM±Dev | FPKM±Dev | change | *p*-value |
| *Se*CHRXVI | 13815 | 15572 | *MAL32* | *YBR299W* | Maltase (EC 3.2.1.20) | 9.9 ±0.2 | 5218.4 ± 365.5 | 525.6 | 0 |
| *Se*CHRV | 569448 | 571205 | *MAL32* | *YBR299W* | Maltase (EC 3.2.1.20) | 9.6 ± 0.0 | 4620.4 ± 384.1 | 481.3 | 0 |
| *Se*CHRV | 566765 | 568606 | *MAL31* | *YBR298C* | Maltose permease (Se*MALT2*) | 5.5 ± 0.4 | 1451.0 ± 39.7 | 262.7 | 0 |
| *Se*CHRXVI | 16413 | 18254 | *MAL31* | *YBR298C* | Maltose permease (*SeMALT4*) | 6.9 ± 0.4 | 1683.8 ± 30.7 | 244.5 | 0 |
| *Se*CHRXIII | 4195 | 5964 | *IMA1* | *YGR287C* | Major isomaltase (alpha-1,6-glucosidase/alpha-methylglucosidase) | 8.4 ± 1.2 | 1671.4 ± 283.7 | 199.9 | 0 |
| *Se*CHRXVI | 21821 | 23590 | *IMA1* | *YGR287C* | Major isomaltase (alpha-1,6-glucosidase/alpha-methylglucosidase) | 3.4 ± 0.7 | 645.2 ± 95.7 | 189.9 | 0 |
| *Se*CHRIV | 971754 | 973529 | *DAK2* | *YFL053W* | Dihydroxyacetone kinase | 19.2 ± 0.6 | 3342.3 ± 171.5 | 174.1 | 0 |
| *Se*CHRII | 8497 | 10338 | *MAL31* | *YBR298C* | Maltose permease (*SeMALT1*) | 0.5 ± 0.0 | 30.8 ± 1.5 | 67.7 | 3.18E-82 |
| *Se*CHRXVI | 25036 | 25626 | YJL218W | YJL218W | Putative acetyltrasferase | 8.3 ± 2.3 | 528.3 ± 4.3 | 63.9 | 2.10E-246 |
| *Se*CHRIV | 968740 | 970695 | *AQY3* | *YFL054C* | Putative channel-like protein; similar to Fps1 | 10.7 ± 2.2 | 529.6 ± 22.0 | 49.3 | 0 |
| *Se*CHRII | 5766 | 7535 | *IMA1* | *YGR287C* | Major isomaltase (alpha-1,6-glucosidase/alpha-methylglucosidase) | 1.6 ± 0.1 | 66.6 ± 5.1 | 41.7 | 2.65E-150 |
| *Se*CHRIV | 974654 | 975250 | *REE1* | *YJL217W* | Cytoplasmic protein involved in the regulation of enolase | 27.0 ± 1.1 | 1079.8 ± 76.4 | 40.0 | 2.34E-293 |
| *Se*CHRX | 326579 | 327577 | *TDH1* | *YJL052W* | Glyceraldehyde-3-phosphate dehydrogenase 1 | 39.1 ± 0.1 | 1196.1 ± 318.7 | 30.6 | 8.19E-188 |
| *Se*CHRV | 564762 | 565970 | *MAL33* | *YBR297W* | MAL-activator protein; part of complex locus MAL3 | 10.0 ± 1.2 | 196.3 ± 17.0 | 19.6 | 4.38E-171 |
| *Se*CHRIV | 512918 | 513952 | *YRO2* | *YBR054W* | Homolog to HSP30 heat shock protein YRO1 | 128.1 ± 6.9 | 2348.5 ± 110.4 | 18.3 | 2.46E-238 |
| *Se*CHRXV | 735045 | 736550 | YNR073C | YNR073C | Mannitol dehydrogenase | 1.8 ± 0.2 | 31.8 ± 8.3 | 17.6 | 8.08E-62 |
| *Se*CHRX | 732395 | 734113 | *HXT13* | *YEL069C* | Hexose transporter | 14.2 ± 3.8 | 202.2 ± 19.0 | 14.3 | 3.41E-113 |
| *Se*CHRIV | 552424 | 553068 | *HSP26* | *YBR072W* | Heat shock protein of 26 kDa, expressed in entry to stationary phase | 137.5 ± 19.5 | 1520.3 ± 33.7 | 11.1 | 1.22E-147 |
| *Se*CHRVII | 585691 | 587421 | *MUP1* | *YGR055W* | High affinity methionine permease | 5.1 ± 1.6 | 55.2 ± 7.4 | 10.8 | 9.30E-67 |
| *Se*CHRVII | 934199 | 934486 | *SPG1* | *YGR236C* | Questionable ORF | 6.3 ± 1.6 | 65.3 ± 5.5 | 10.3 | 2.33E-31 |
| *Se*CHRXIII | 942285 | 943424 | *FDH1* | *YOR388C* | Protein with similarity to formate dehydrogenases | 5.9 ± 0.6 | 53.3 ± 3.1 | 9.1 | 2.94E-64 |
| *Se*CHRXVI | 829072 | 830475 | *TDA6* | *YPR157W* | Putative vacuolar protein sorting-associated | 7.9 ± 0.0 | 71.2 ± 4.8 | 9.0 | 3.39E-86 |
| *Se*CHRX | 727746 | 728780 | YNR071C | YNR071C | Uncharacterized isomerase | 90.7 ± 1.1 | 814.2 ± 27.6 | 9.0 | 5.25E-145 |
| *Se*CHRVII | 23171 | 24301 | YNL134C | YNL134C | Uncharacterized protein | 15.7 ± 4.2 | 131.7 ± 20.3 | 8.4 | 2.61E-64 |
| *Se*CHRV | 81046 | 82548 | *YEF1* | *YEL041W* | ATP-NADH kinase; phosphorylates both NAD and NADH | 28.6 ± 1.9 | 233.5 ± 15.0 | 8.2 | 1.32E-114 |
| *Se*CHRXIII | 669178 | 670125 | YMR206W | YMR206W | Uncharacterized protein | 2.2 ± 0.3 | 17.0 ± 6.7 | 7.8 | 2.05E-19 |
| *Se*CHRXIV | 330291 | 331349 | *YGP1* | *YNL160W* | Secreted glycoprotein produced in response to nutrient limitation | 745.5 ± 120.3 | 5588.8 ±487.2 | 7.5 | 6.62E-94 |
| *Se*CHRV | 468857 | 469288 | *SPI1* | *YER150W* | Strong similarity to putative cell surface glycoprotein Sed1p | 233.5 ± 74.6 | 1727.5 ±163.2 | 7.4 | 1.79E-58 |
| *Se*CHRXIV | 685860 | 686156 | YNR034W-A | YNR034W-A | Uncharacterized protein | 1188.2 ± 537.1 | 8530.4 ± 862.4 | 7.2 | 1.04E-09 |
| *Se*CHRV | 264185 | 265087 | *PIC2* | *YER053C* | Mitochondrial copper and phosphate carrier | 129.1 ± 24.2 | 920.6 ± 57.0 | 7.1 | 4.21E-84 |
| *Se*CHRXIII | 9572 | 11416 | *MAL31* | *YBR298C* | Maltose permease (*SeMALT3*) | 29.9 ± 1.1 | 204.6 ± 30.1 | 6.8 | 5.22E-84 |
| *Se*CHRXIII | 935354 | 937039 | *STL1* | *YDR536W* | Sugar transporter-like protein | 4.0 ± 1.2 | 26.6 ± 1.2 | 6.6 | 7.96E-40 |
| *Se*CHRXI | 300992 | 301213 | YKL065W-A | YKL065W-A | Uncharacterized protein | 7.3 ± 3.0 | 48.0 ± 4.9 | 6.6 | 1.94E-15 |
| *Se*CHRVII | 1050057 | 1051673 | *YPS6* | *YIR039C* | GPI-anchored aspartic protease | 2.7 ± 0.8 | 17.5 ± 3.6 | 6.5 | 1.92E-27 |
| *Se*CHRXIV | 365428 | 366558 | YNL134C | YNL134C | NADH-dependent aldehyde reductase | 68.1 ± 11.1 | 440.1 ±52.0 | 6.5 | 3.99E-75 |
| *Se*CHRV | 298146 | 298631 | *RGI1* | *YER067W* | Protein of unknown function; involved in energy metabolism | 769.0 ± 77.4 | 4755.0 ± 13.1 | 6.2 | 1.39E-99 |
| *Se*CHRII | 353374 | 355188 | *BAP3* | *YDR046C* | Valine transporter | 7.9 ± 0.3 | 48.1 ± 0.2 | 6.1 | 3.65E-62 |
| *Se*CHRII | 326147 | 327121 | *MRH1* | *YDR033W* | Strong similarity to putative heat shock protein YRO2 | 541.4 ± 26.6 | 3171.9 ±283.0 | 5.9 | 7.70E-81 |
| *Se*CHRX | 8474 | 9253 | YKL071W | YKL071W | Putative protein of unknown function | 6.1 ± 0.7 | 33.0 ± 3.0 | 5.4 | 4.48E-27 |
| *Se*CHRXVI | 223023 | 224216 | *OYE3* | *YPL171C* | NAD(P)H dehydrogenase | 39.1 ± 1.3 | 210.0 ± 15.0 | 5.4 | 1.21E-72 |
| *Se*CHRIII | 151700 | 152689 | *HSP30* | *YCR021C* | Heat shock protein located in the plasma membrane | 23.1 ± 3.9 | 122.2 ± 5.3 | 5.3 | 8.78E-53 |
| *Se*CHRVIII | 324960 | 325898 | *GCY1* | *YOR120W* | Similar to mammalian aldo keto reductases | 192.6 ± 73.2 | 995.6 ± 14.7 | 5.2 | 2.35E-35 |
| *Se*CHRXIII | 373028 | 373924 | YLL056C | YLL056C | Putative protein of unknown function | 21.8 ± 4.8 | 111.5 ± 13.4 | 5.1 | 1.52E-40 |
| *Se*CHRI | 199000 | 199770 | YKL071W | YKL071W | Putative protein of unknown function | 11.4 ± 0.7 | 56.9 ± 5.9 | 5.0 | 2.18E-34 |
| *Se*CHRII | 488814 | 489014 | *COX26* | *YDR119W-A* | Stabilizes or regulates formation of respiratory chain complexes | 46.7 ± 5.3 | 228.4 ± 13.9 | 4.9 | 8.40E-34 |
| *Se*CHRI | 60828 | 61628 | YAL037W | YAL037W | Putative protein of unknown function | 5.4 ± 1.1 | 26.1 ± 0.1 | 4.8 | 1.01E-20 |
| *Se*CHRXIII | 923767 | 925488 | *HXT13* | *YEL069C* | Putative transmembrane polyol transporter | 61.6 ± 0.7 | 294.3 ± 33.3 | 4.8 | 4.58E-63 |
| *Se*CHRX | 594900 | 595748 | YJR096W | YJR096W | Xylose and arabinose reductase | 34.4 ± 6.4 | 162.8 ± 8.6 | 4.7 | 6.20E-46 |
| *Se*CHRXI | 567594 | 568697 | *ECM4* | *YKR076W* | ExtraCellular Mutant | 33.5 ± 6.2 | 157.1 ± 7.3 | 4.7 | 9.69E-48 |
| *Se*CHRVIII | 434753 | 437188 | *GAC1* | *YOR178C* | Regulatory subunit for Glc7p | 14.5 ± 1.2 | 67.2 ± 5.7 | 4.6 | 7.52E-54 |
| *Se*CHRXVI | 897255 | 897722 | *HPA2* | *YPR193C* | Histone and other Protein Acetyltransferase | 17.7± 1.8 | 81.3 ± 11.3 | 4.6 | 7.43E-28 |
| *Se*CHRXII | 807640 | 808032 | YLR297W | YLR297W | Protein of unknown function | 71.3 ± 4.3 | 326.6 ± 0.9 | 4.6 | 1.67E-50 |
| *Se*CHRII | 127205 | 127621 | *MOH1* | *YBL049W* | Protein of unknown function, essential for stationary phase survival | 31.6± 1.8 | 143.0 ± 1.0 | 4.5 | 4.00E-38 |
| *Se*CHRVII | 289727 | 290107 | *GPG1* | *YGL121C* | Proposed gamma subunit of the heterotrimeric G protein | 293.9 ± 26.0 | 1275.5 ±173.3 | 4.3 | 3.89E-48 |
| *Se*CHRXVI | 625902 | 629342 | *CSR2* | *YPR030W* | Similarity to YBL101c | 11.6 ± 1.5 | 50.1 ± 5.3 | 4.3 | 7.74E-46 |
| *Se*CHRIV | 542614 | 543234 | *TIP1* | *YBR067C* | Cell wall mannoprotein | 131.0 ± 33.5 | 567.5 ± 138.0 | 4.3 | 1.09E-26 |
| *Se*CHRV | 455522 | 456415 | *MAG1* | *YER142C* | 3-methyladenine DNA glycosylase | 14.3 ± 1.0 | 61.9 ± 7.2 | 4.3 | 2.97E-32 |
| *Se*CHRVII | 949163 | 949600 | *FMP43* | *YGR243W* | Highly conserved subunit of the mitochondrial pyruvate carrier | 15.4 ± 2.3 | 66.5 ± 1.1 | 4.3 | 2.48E-24 |
| *Se*CHRXI | 8566 | 10413 | *JEN1* | *YKL217W* | Carboxylic acid transporter protein homolog | 5.2 ± 0.6 | 22.0 ± 0.1 | 4.3 | 1.70E-29 |
| *Se*CHRVII | 10469 | 11500 | YNR071C | YNR071C | Aldose 1-epimerase | 485.0 ± 46 | 2021.5 ± 30.5 | 4.2 | 7.63E-62 |
| *Se*CHRIX | 397417 | 398181 | *NRE1* | *YIR035C* | Putative cytoplasmic short-chain dehydrogenase reductase | 154.4 ± 11.9 | 642.0 ± 48.6 | 4.2 | 4.42E-51 |
| *Se*CHRIV | 706015 | 707070 | *ADH5* | *YBR145W* | Alcohol dehydrogenase isoenzyme V | 75.6 ± 0.3 | 312.9 ± 15.6 | 4.1 | 5.42E-57 |
| *Se*CHRXIII | 200932 | 202473 | *RRN11* | *YML043C* | Component of rDNA transcription factor CF | 20.2 ± 1.6 | 5.0 ± 0.5 | -4.0 | 1.56E-21 |
| *Se*CHRXV | 116156 | 119050 | *MSH2* | *YOL090W* | MutS homolog encoding major mismatch repair activity | 22.2 ± 3.4 | 5.5 ± 0.6 | -4.0 | 6.56E-26 |
| *Se*CHRII | 232378 | 232776 | *HTA1* | *YDR225W* | Histone H2A (HTA1 and HTA2 code for nearly identical proteins) | 6216.1 ± 95.8 | 1543.9 ±188.1 | -4.0 | 1.25E-45 |
| *Se*CHRX | 68803 | 71355 | *SWE1* | *YJL187C* | Protein kinase homolog | 22.6 ± 1.2 | 5.6 ± 1.1 | -4.0 | 1.12E-26 |
| *Se*CHRXVI | 305949 | 306761 | *HHO1* | *YPL127C* | Histone H1 | 136.2 ± 7.3 | 33.6 ± 5.4 | -4.1 | 1.57E-35 |
| *Se*CHRXII | 38986 | 39858 | *AQY1* | *YPR192W* | Similarity to plasma membrane and water channel proteins | 54.1 ± 14.2 | 13.3 ± 1.5 | -4.1 | 6.38E-21 |
| *Se*CHRXIV | 714569 | 716618 | *NOG2* | *YNR053C* | Strong similarity to human breast tumor associated autoantigen | 96.0 ± 14.2 | 23.0 ± 3.1 | -4.2 | 3.41E-34 |
| *Se*CHRXI | 164418 | 165815 | *LTV1* | *YKL143W* | Low temperature viability protein | 28.9 ± 4.9 | 6.9 ± 0.3 | -4.2 | 5.77E-23 |
| *Se*CHRXIV | 568431 | 568742 | *HHF1* | *YBR009C* | Histone H4; core histone protein required for chromatin assembly | 6315.8 ± 13.1 | 1489.5 ± 91.2 | -4.2 | 3.61E-58 |
| *Se*CHRXIII | 875200 | 876345 | *SCW10* | *YMR305C* | Member of the glucanase gene family | 209.0 ±25.4 | 48.6 ± 6.7 | -4.3 | 1.61E-38 |
| *Se*CHRII | 519002 | 519394 | *CCW22* | *YDR134C* | Cell wall protein; YDR134C has a paralog, | 4393.2 ±171.7 | 1003.3 ± 25.8 | -4.4 | 4.41E-65 |
| *Se*CHRVII | 850827 | 852332 | *CRH1* | *YGR189C* | Similarity to *Aspergillus fumigatus* rAsp | 475.8 ± 41.7 | 108.6 ± 6.6 | -4.4 | 4.86E-52 |
| *Se*CHRXII | 657361 | 658839 | *TUB4* | *YLR212C* | Gamma tubulin-like protein, interacts with Spc98p and Spc97p | 44.5 ± 1.7 | 10.1 ± 1.7 | -4.4 | 1.19E-32 |
| *Se*CHRIII | 185259 | 186302 | *FEN1* | *YCR034W* | Probable subunit of 1,3-beta-glucan synthase\; homolog of ELO1 | 377.5 ± 20.0 | 85.3 ± 10.5 | -4.4 | 2.04E-46 |
| *Se*CHRXI | 249340 | 250086 | *CWP1* | *YKL096W* | Cell wall mannoprotein | 1334.2 ± 125.2 | 299.6 ± 33.9 | -4.5 | 3.55E-55 |
| *Se*CHRXIV | 380345 | 382882 | *SPC98* | *YNL126W* | Spindle pole body component | 20.1 ± 0.2 | 4.5 ± 0.6 | -4.5 | 4.10E-16 |
| *Se*CHRII | 153948 | 156062 | *POL12* | *YBL035C* | B subunit of DNA polymerase alpha-primase complex | 18.1 ± 4.4 | 4.0 ± 0.1 | -4.5 | 3.34E-21 |
| *Se*CHRIX | 113174 | 114583 | *SIM1* | *YIL123W* | Involved in cell cycle regulation and aging | 287.9 ± 23.8 | 63.7 ± 7.6 | -4.5 | 1.55E-48 |
| *Se*CHRXII | 431462 | 432901 | *PUT1* | *YLR142W* | Proline oxidase (proline dehydrogenase) | 19.0 ± 2.6 | 4.2 ± 1.2 | -4.5 | 1.27E-19 |
| *Se*CHRXV | 416676 | 417626 | *GIC1* | *YHR061C* | GTPase-interacting component 1 | 61.3 ± 3.4 | 13.5 ± 0.6 | -4.5 | 6.35E-36 |
| *Se*CHRIX | 281450 | 284902 | *IRR1* | *YIL026C* | Subunit of the cohesin complex | 18.3 ± 3.7 | 4.0 ± 0.1 | -4.6 | 4.53E-28 |
| *Se*CHRV | 216592 | 217746 | *MIG3* | *YER028C* | Transcriptional regulator | 32.4 ± 0.1 | 7.1 ± 0.2 | -4.6 | 4.56E-29 |
| *Se*CHRXIII | 690119 | 691672 | *GAS3* | *YMR215W* | Similarity to GAS1 protein | 84.6 ± 7.6 | 18.4 ± 0.3 | -4.6 | 3.51E-49 |
| *Se*CHRV | 78890 | 80452 | *GDA1* | *YEL042W* | Guanosine diphosphatase located in the Golgi | 125.7 ± 3.4 | 27.0 ± 1.6 | -4.7 | 8.91E-55 |
| *Se*CHRVIII | 723692 | 724654 | YOR342C | YOR342C | Protein of unknown function | 76.3 ± 14.1 | 16.0 ± 0.9 | -4.8 | 6.75E-32 |
| *Se*CHRXI | 338942 | 340531 | *PRI2* | *YKL045W* | p58 polypeptide of DNA primase | 24.6 ± 3.6 | 5.2 ± 1.1 | -4.8 | 1.36E-24 |
| *Se*CHRIV | 356746 | 357555 | *KNH1* | *YDL049C* | KRE9 homolog | 44.9 ± 2.5 | 9.4 ± 1.2 | -4.8 | 2.34E-28 |
| *Se*CHRII | 478219 | 479349 | *PDS1* | *YDR113C* | 42-kDa nuclear protein | 57.9 ± 2.9 | 12.1 ± 0.1 | -4.8 | 1.49E-38 |
| *Se*CHRVIII | 737587 | 739464 | *PUT4* | *YOR348C* | Putative proline-specific permease | 16.3 ± 5.7 | 3.4 ± 0.5 | -4.8 | 2.18E-18 |
| *Se*CHRXIII | 225859 | 227010 | *YOX1* | *YML027W* | Homeobox-domain containing protein | 67.2 ± 7.6 | 13.9 ± 1.0 | -4.8 | 2.01E-40 |
| *Se*CHRXV | 257246 | 258829 | YOL019W | YOL019W | Protein of unknown function | 53.1 ± 0.5 | 10.9 ± 0.3 | -4.9 | 2.32E-45 |
| *Se*CHRIX | 70415 | 72874 | *AXL2* | *YIL140W* | Localizes to the plasma membrane | 25.0 ± 3.8 | 5.1 ± 0.2 | -4.9 | 2.83E-33 |
| *Se*CHRIV | 272749 | 274293 | *DUN1* | *YDL101C* | Protein kinase | 37.2 ± 2.2 | 7.5 ± 0.7 | -4.9 | 7.02E-37 |
| *Se*CHRVII | 441348 | 441710 | *CGR1* | *YGL029W* | Weak similarity to human chromatin assembly factor I p150 chain | 234.1 ± 43.3 | 46.9 ± 1.9 | -5.0 | 6.22E-35 |
| *Se*CHRXIV | 707536 | 708876 | *LYS9* | *YNR050C* | Saccharopine dehydrogenase (NADP+, L-glutamate forming) | 262.6 ± 6.6 | 50.6 ± 2.2 | -5.2 | 1.52E-72 |
| *Se*CHRXI | 236917 | 241500 | *HSL1* | *YKL101W* | Putative protein kinase homologous to S. pombe cdr1 nim1 | 24.6 ± 4.8 | 4.7 ± 1.0 | -5.2 | 9.62E-34 |
| *Se*CHRXIII | 545844 | 546851 | *FDO1* | *YMR144W* | Protein involved in directionality of mating type switching | 33.0 ± 5.3 | 6.3 ± 0.7 | -5.2 | 3.51E-26 |
| *Se*CHRXVI | 364893 | 366263 | *EEB1* | *YPL095C* | Acyl-coenzymeA:ethanol O-acyltransferase | 132.0 ± 6.5 | 25.1 ± 0.7 | -5.3 | 1.03E-60 |
| *Se*CHRIV | 597086 | 597862 | *POL30* | *YBR088C* | Profilerating cell nuclear antigen (PCNA) | 257.7 ± 24.3 | 48.0 ± 2.9 | -5.4 | 2.48E-39 |
| *Se*CHRX | 48344 | 50713 | *ACO2* | *YJL200C* | Putative mitochondrial aconitase isozyme | 115.6 ± 6.0 | 21.3 ± 3.1 | -5.4 | 1.56E-58 |
| *Se*CHRX | 463176 | 465695 | *RBH2* | *YJR030C* | Putative protein of unknown function | 19.5 ± 3.3 | 3.6 ± 0.6 | -5.4 | 2.75E-31 |
| *Se*CHRVI | 246194 | 247399 | *IRC7* | *YFR055W* | Beta-lyase involved in the production of thiols | 38.6 ± 4.2 | 7.1 ± 0.7 | -5.4 | 1.46E-34 |
| *Se*CHRII | 233489 | 233884 | *HTB2* | *YBL002W* | Histone H2B (HTB1 and HTB2 code for nearly identical proteins) | 5683.3 ± 69.9 | 1042.7 ±133.2 | -5.5 | 1.39E-66 |
| *Se*CHRVIII | 240512 | 241426 | *CDC21* | *YOR074C* | Thymidylate synthase | 54.8 ± 9.2 | 10.0 ± 0.9 | -5.5 | 7.46E-33 |
| *Se*CHRXVI | 238727 | 239494 | *SVS1* | *YPL163C* | Serine and threonine rich protein. | 158.6 ± 18.0 | 28.5 ± 7.3 | -5.6 | 2.70E-39 |
| *Se*CHRXIV | 406937 | 409589 | *DBP2* | *YNL112W* | TP-dependent RNA helicase of DEAD box family | 84.8 ± 24.1 | 14.8 ± 2.4 | -5.7 | 1.02E-35 |
| *Se*CHRV | 305067 | 307733 | *RNR1* | *YER070W* | Ribonucleotide reductase | 177.1 ± 51.7 | 30.8 ± 7.7 | -5.8 | 4.36E-09 |
| *Se*CHRXII | 955392 | 956429 | *SUR4* | *YLR372W* | Required for conversion of 24-carbon fatty acids to 26-carbon species | 387.3 ± 53.3 | 65.7 ± 2.1 | -5.9 | 1.30E-62 |
| *Se*CHRXVI | 62522 | 64180 | *CLN2* | *YPL256C* | G(sub)1 cyclin | 46.3 ± 8.4 | 7.3 ± 1.0 | -6.3 | 1.10E-41 |
| *Se*CHRXIII | 917562 | 919325 | *SAM3* | *YPL274W* | Strong similarity to amino-acid transport proteins | 21.3 ± 0.0 | 3.4 ± 0.2 | -6.3 | 1.04E-38 |
| *Se*CHRXI | 500540 | 502345 | *GAP1* | *YKR039W* | General amino acid permease, proton symport transporter | 773.1± 88.6 | 121.7 ± 4.2 | -6.4 | 6.16E-92 |
| *Se*CHRXIV | 83384 | 84223 | *PCL1* | *YNL289W* | G(sub)1 cyclin that associates with PHO85 | 278.8 ± 4.8 | 41.8 ± 2.8 | -6.7 | 9.08E-86 |
| *Se*CHRXV | 280167 | 281201 | *CSI2* | *YOL007C* | Structural component of the chitin synthase 3 complex | 116.9 ± 11.8 | 16.1 ± 1.3 | -7.3 | 9.17E-66 |
| *Se*CHRXI | 212836 | 213984 | *RAD27* | *YKL113C* | 42 kDa 5' to 3' exonuclease required for Okazaki fragment processing | 65.1 ± 8.9 | 8.9 ± 0.9 | -7.3 | 3.20E-52 |
| *Se*CHRXII | 612599 | 614092 | *TOS4* | *YLR183C* | Similarity to YDR501w | 61.9 ± 8.5 | 8.0 ± 0.3 | -7.7 | 4.04E-60 |
| *Se*CHRVIII | 196815 | 198143 | *STD1* | *YOR047C* | Dosage-dependent modulator of glucose repression | 45.5 ± 0.4 | 5.8 ± 1.2 | -7.8 | 1.10E-51 |
| *Se*CHRIV | 441107 | 442810 | *MCD1* | *YDL003W* | Mitotic omosome Determinant; similar to *S. pombe RAD21* | 61.2 ± 14.1 | 7.5 ± 1.0 | -8.2 | 1.54E-51 |
| *Se*CHRXIV | 61224 | 61532 | *TOS6* | *YNL300W* | Hypothetical protein | 756.5 ± 28.8 | 82.8 ± 41.7 | -9.1 | 2.20E-09 |
| *Se*CHRIV | 73671 | 74813 | YDL211C | YDL211C | Protein of unknown function | 36.0 ± 2.8 | 3.4 ± 0.2 | -10.7 | 1.21E-53 |
| *Se*CHRXI | 493149 | 493970 | *DAL80* | *YKR034W* | Negative regulator of multiple nitrogen catabolic genes | 25.8 ± 4.4 | 2.1 ± 0.6 | -12.3 | 8.69E-34 |
| *Se*CHRII | 925500 | 930622 | *HXT3* | *YDR345C* | Low-affinity glucose transporter | 541.0 ± 73.3 | 34.7 ± 1.8 | -15.6 | 1.55E-163 |
| *Se*CHRVII | 692475 | 693617 | *CLB6* | *YGR109C* | B-type cyclin | 21.3 ± 3.7 | 1.2 ± 0.7 | -17.6 | 7.11E-37 |
| *Se*CHRVII | 892340 | 893290 | *RTA1* | *YGR213C* | Involved in 7-aminocholesterol resistance | 82.9 ± 1.1 | 2.5 ± 0.8 | -33.2 | 2.70E-106 |

**Figure S8: Comparison of divergent promoter elements located near maltose transporter genes at *MAL* loci of *S. cerevisiae* and *S. eubayanus* CBS 12357^T^.**

**
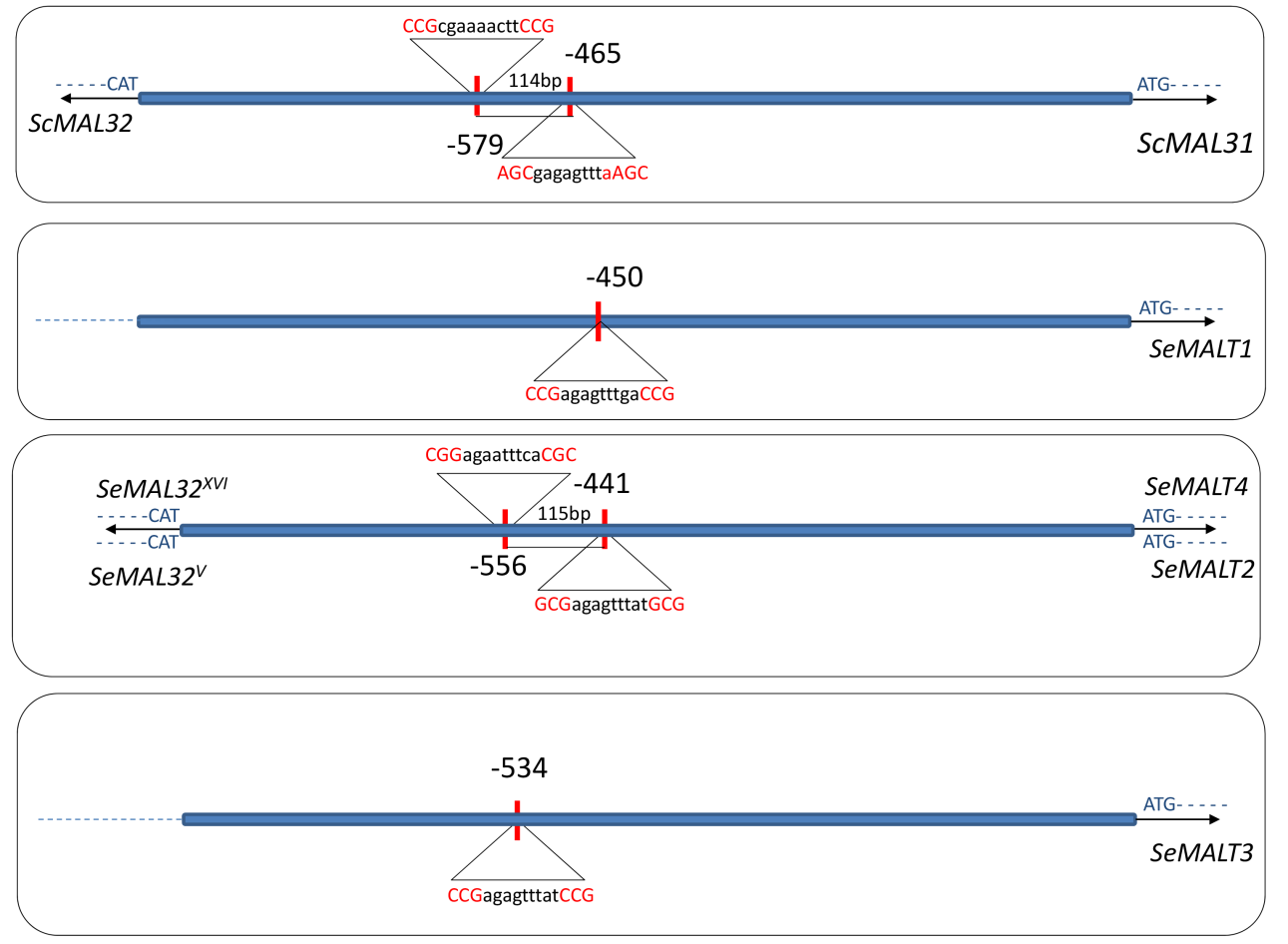
**

**Figure S9: Characterization of the *S. cerevisiae* strains (●) IMZ616 (Marques *et al.* 2018), (○) IMX1253 (Sc*TEF1_pr_-SeMALT1-ScCYC1_ter_*), (▲) IMX1254 (Sc*TEF1_pr_-SeMALT2-ScCYC1_ter_)*, (△) IMX1255 (Sc*TEF1_pr_-SeMALT3-ScCYC1_ter_*), (●) IMX1365 (Sc*TEF1_pr_-ScMAL11-ScCYC1_ter_*) and (●) *S. eubayanus* CBS 12357^T^ for growth on maltotriose**. The strains were grown in SM_U_G and SM_U_M at 20 ^o^C. Growth on glucose (**A**) and on maltotriose (**C**) was monitored based on optical density measurements at 660 nm (OD_660nm_). Concentrations of glucose (**B**) and maltotriose (**D**) in culture supernatants were measured by HPLC. Data are presented as average and standard deviation of three biological replicates.


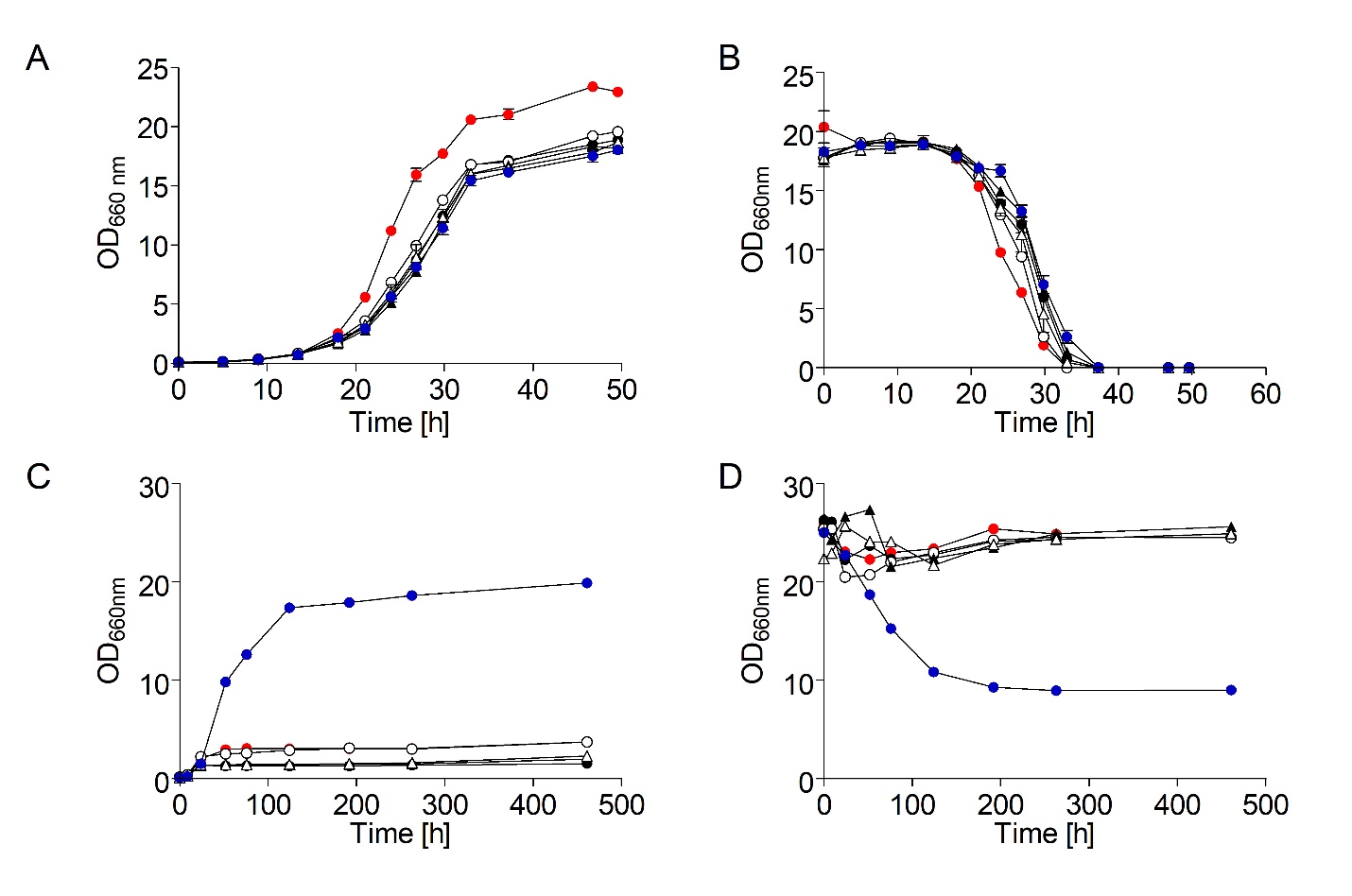


Marques WL, Mans R, Henderson RK *et al.* Combined engineering of disaccharide transport and phosphorolysis for enhanced ATP yield from sucrose fermentation in *Saccharomyces cerevisiae*. *Metab Eng* 2018;**45**: 121-33.
